# Environmental Factors Associated with Success Rates of Australian Stock Herding Dogs

**DOI:** 10.1371/journal.pone.0104457

**Published:** 2014-08-19

**Authors:** Elizabeth R. Arnott, Jonathan B. Early, Claire M. Wade, Paul D. McGreevy

**Affiliations:** Faculty of Veterinary Science, University of Sydney, Sydney, Australia; University of Missouri, United States of America

## Abstract

This study investigated the current management practices associated with stock herding dogs on Australian farms. A parallel goal was to determine whether these practices and the characteristics of the dog handlers were associated with success rates. Success rate refers to the proportion of dogs acquired by the farmer that were retained as working dogs. Data on a total of 4,027 dogs were obtained through The Farm Dog Survey which gathered information from 812 herding dog owners around Australia. Using logistic regression, significant associations were identified between success rate and seven variables: dog breed, housing method, trial participation, age of the dog at acquisition, electric collar use, hypothetical maximum treatment expenditure and the conscientiousness score of the owner's personality. These findings serve as a guide to direct further research into ways of optimising herding dog performance and welfare. They emphasise the importance of not only examining the genetic predispositions of the working dog but also the impact the handler can have on a dog's success in the workplace.

## Introduction

The Australian cattle and sheep industries function in a climate of increasing input costs, competition with subsidised international markets and variable commodity prices [1]. To maintain profitability, producers have had to invest in various methods to improve productivity. Investments have occurred in areas such as livestock genetics [2], pasture improvement [3] and marketing [4]–[6]. With an estimated 270,000 stock herding dogs working in rural Australia [7], [8], these animals represent a significant component of the labour force in the livestock industries. Therefore, a similar investment to optimise their performance and efficiency may be warranted.

Australia has 91,000 livestock producers [8], who employ an average of three to four working dogs [7], [9]. It is currently impossible to quantify the number of herding dogs bred and the proportion that are successful. It has been estimated that an average of 25% of working dogs recruited for training in Australia fail to graduate successfully [10]. The cost associated with acquiring, keeping and training an unsuccessful herding dog for twelve months, prior to its eventual dismissal, has been estimated to be in excess of AU$1,000 [11]. This degree of performance failure represents costly wastage.

Behavioural issues are the leading cause of performance failure of dogs across several working sectors [10], [12], [13]. Addressing this so-called behavioural wastage demands a focus on both the behavioural genetics of these dogs and on the environmental influences that affect behaviour. There is a growing body of evidence that canine learning and welfare are significantly influenced by husbandry practices and training methodology [14]–[17]. Furthermore, research examining working dogs in the police and military sectors indicates that individual handler characteristics and their relationship with their dog may have an effect on performance outcomes [15], [18].

Identifying factors associated with stock herding dog success and failure will enable producers to adapt their practices to gain maximum financial return from their dogs. However, the possible incentive to reduce cull rates of dogs is not limited to profit maximisation. In the sustainable agriculture paradigm, farming practices must be socially responsible as well as economically viable to sustain productivity over time [19]. Thus, the impetus to optimise the management of farm dogs should be to respond to the growing public awareness of the welfare issues associated with food production. In Australia, for example, the economics related to public opinion have had consequences for producers of export cattle [20], wool [21] and eggs [22]. In recent times, proposed changes to codes of practice that impact Australian stock herding dogs have caused controversy and disagreement among stakeholders [23]. Therefore, objective information is required to establish what may be considered appropriate care of stock herding dogs to safe-guard their welfare.

This paper reports the findings of the Australian Farm Dog Survey. The questionnaire was designed to explore the current canine management and training practices on Australian farms and the characteristics of the farmers who handle and breed the working dogs. These variables were analysed to explore potential risk factors for herding dog failure.

## Materials and Methods

### Ethics Statement

Approval for this study was granted from the University of Sydney Human Research Ethics Committee (Approval number 15474).

### The Questionnaire

Prior to publication of the questionnaire, advice was sought from members of the Working Kelpie Council of Australia to ensure that the question terminology was appropriate for the target audience. A pilot distribution of the survey to 125 solicited participants led to some minor modifications prior to widespread distribution.

The online version of the Farm Dog Survey was administered for a three-month period from 10 March 2013 to 10 June 2013. All promotional materials indicated that a hard copy of the survey with a reply paid envelope could be provided to participants on request.

The target population for the survey was all stock herding dog users in Australia. Participation was encouraged with an incentive in the form of the opportunity to win commercial working dog food in a prize draw at the end of the survey period. An introductory message gave participants the option to respond anonymously and the assurance of confidentiality if they chose to leave their details to enter the prize draw.

A link to the online questionnaire was posted on the websites of the University of Sydney, Meat and Livestock Australia and the Working Kelpie Council of Australia. It was advertised through features in multiple rural newspapers, on two national television programmes and in two agricultural magazines with Australia-wide distributions. The committee of the 2013 Casterton Kelpie Auction (one of Australia's leading working dog auction events) promoted the survey in a mail-out to past and present vendors and purchasers. The researchers also recruited survey participants, in person, at herding dog trials during the study period.

The online version of the Farm Dog Survey was constructed using the survey system Qsmart (Torque Management Systems Limited, Auckland, New Zealand). The entire questionnaire had a maximum of 143 items divided into 10 sections. However, participants had fewer questions to answer if they responded in the negative to questions about certain activities, such as breeding or trialling of dogs. Furthermore, the participants had the option in three sections of the questionnaire to give details on up to three of their dogs. Choosing to answer these questions for one or two dogs reduced the number of questions to be answered by 28 or 56, respectively. The logic system of the online survey allowed for the redirection of participants to questions of relevance. Sections 8 and 9 of the questionnaire asked participants to indicate the value they placed on various physical and behavioural traits in the working dog and were not relevant to the current report. The remaining eight sections are summarised below. For the complete questionnaire see Questionnaire S1.

Section 1 gathered details on the size and location of the respondent's main property, the numbers and types of livestock produced and the number of working dogs used.

Section 2 asked the respondent to give details on one to three of the dogs they currently work with most often. These details included the dogs' signalment, usage, origin and purchase cost, housing, recent veterinary expenses and trial participation and performance.

Section 3 was available to those participants who reported that they bred working dogs. Questions related to the breed and number of dogs in their breeding program, the purpose and aims of their breeding program, the degree of in-breeding they are willing to employ and the age and price of their pups at sale.

Section 4 investigated the workload of the dogs by asking their owners, “at peak times, how much time does your top dog spend working on average, each day and each week?” Respondents could select “less than two hours”, “two to four hours”, “four to six hours” or “more than six hours” per day and from one to seven days per week. In addition, they were asked how often their dogs were exercised (including time spent off the chain or out of the cage) during off-peak periods. The response options were; “less than weekly”, “weekly”, “twice weekly”, “three to five times each week”, “daily” and “at least twice daily”.

Section 5 asked for the reason, destination and age of dismissal of up to three dogs that the respondent had stopped working with due to failure and due to retirement. They were asked to report the percentage of the dogs they acquire or retain for work that become successful working dogs. The options were “less than 50%”, “50–64%”, “65–79%”, “80–99%” and “100%”.

Section 6 was modelled on Section 4 of the Australian Animal Welfare Strategy (AAWS) Working Dog Survey [10]. Questions related to the method and equipment used to train stock herding dogs and the dog-training education of the respondent. Respondents were categorised as using positive reinforcement if they described using “food treats”, “patting” or “verbal praise” when training. In addition, respondents were asked “how much time is spent with the dog during an average training session?” The options were; “I don't have formal training sessions”, “less than 15 minutes”, “15–30 minutes”, “30–60 minutes” and “greater than one hour”. Respondents were also asked to select how many training sessions they give per month from the options: “I don't have formal training sessions”, “less than eight”, “eight to 15”, “16–30” and “more than 30”.

Section 7 asked the respondents to estimate the yearly routine costs of owning a working dog and, secondly, what they would be willing to spend on their best dog to allow it to return to work from illness or injury. They could choose a response from one of six categories ranging from “AU$200 or less” to “more than AU$5,000”.

Section 10 requested basic demographic information from the respondents but also asked them to choose one of four descriptions to reflect their general attitude towards, and perception of, their working dogs. These were; “companion”, “workmate”, “employee” and “a workplace resource only”. Finally, the survey contained the ten-item Big Five Inventory (BFI-10) human personality test which consists of 10 short-phrase items rated on a five-point scale from “disagree strongly” to “agree strongly” [24]. The BFI – 10 is an abbreviated version of the 44 item Big Five Inventory (BFI-44) which is designed to measure and describe personality in terms of the five personality dimensions ‘neuroticism’, ‘extraversion’, ‘openness’, ‘agreeableness’ and ‘conscientiousness’. As the validity of the measurement of the agreeableness trait has been found to suffer in the abridged version of the BFI, an additional item was added to the test to measure this trait [24]. A twelfth phrase, taken from the BFI-44, was added to further assess the openness trait. This was decided after consultation with the Working Kelpie Council members who believed that for the population in question, Australian livestock producers, openness to ideas and actions was more relevant than openness to fantasy and aesthetics. Without the addition of the twelfth item, the BFI-10 assesses openness with an emphasis on fantasy and aesthetics rather than ideas and actions [24]. Participants were scored from one (low expression) to five (high expression) for each of the five personality traits according to their average ratings on the twelve statements.

### Calculations and Analysis

The outcome variable of interest was termed “success rate”. This was defined as the percentage of the dogs acquired by respondents for training or immediate use as a herding dog that ultimately became successful working dogs. The converse of the success rate was considered the “cull rate”. As the survey respondents selected from success ranges, for example 80–99%, the midpoints of the ranges were used to calculate the overall mean success rate. To achieve adequate sample sizes within the levels of the variables and to allow for meaningful comparisons between outcomes, the success variable was collapsed to a binary outcome; below average success and average or above average success.

Logistic regression was used to compare the respondents' reported success rates with 22 variables that related to their gender, age, personality and view of their dog, their dog training methodology and experience, their involvement in breeding and breeding practices, the work demands on their dogs during peak periods, the frequency of exercise they provide their dogs and the number of dogs they own.

A second logistic regression was performed to compare the reported success rates with 11 variables describing the dogs the respondents currently work with most often. These variables related to the dogs' breed and sex, their origin, cost, age and training level at acquisition, the type of work they perform, participation in working dog trials, housing and veterinary expenses. Logistic regression was used for this analysis as every generalized linear mixed model analysis that included owner as a random factor failed to converge.

In both cases, the logistic regression initially contained a full model and used stepwise backward elimination. The least significant dependent variables were removed from the model. The Wald test was used for dropping terms and significance was set at p<0.05. The Wald test is equivalent to an F statistic when the data are balanced but is a standard statistic for unbalanced data such as these. For the significant explanatory variables remaining in the final model, the means of their fitted values were calculated to measure the mean probability of average or above average success.

Additionally, the variables dropped from the models were tested for significant associations with success rates (p<0.05) by chi-squared analysis.

Analysis was performed using the program Genstat, 16^th^ Edition (VSN International Ltd, Hemmel Hempstead, UK).

## Results

### The Sample

Eight hundred and twelve responses were received of which 98.6% were online submissions. The respondents submitted details for 1,806 of the dogs currently working, 864 dogs they had most recently dismissed and 1,357 dogs they had most recently retired.

### Canine Success and Failure

The mean success rate reported by survey respondents (n = 812) was 80% (SE = 1.4%). Table 1 shows the range of success rates reported by respondents.

**Table 1:** Estimates by Farm Dog Survey respondents of the percentage of dogs they acquire for stock herding work that become successful working dogs.

Success category	Success Rate	Number of responses (%)
Below average success (<80%)	Less than 50%	60 (7.4)
	50–64%	87 (10.7)
	65–79%	158 (19.5)
Average and above success (≥80%)	80–99%	364 (44.8)
	100%	143 (17.6)

For the 864 dogs most recently failed by respondents, 89 per cent were for non-health related problems. Table 2 details respondents' reasons for dismissing the dogs.

**Table 2:** Reasons Farm Dog Survey respondents failed dogs (n = 864) that they acquired for stock work.

Dismissal reason category	Reason for dismissal	Number of dogs (%)
Behavioural reasons	Lack of working instinct/natural ability	469 (54.3)
	Temperament problems	223 (25.8)
	Training problems	79 (9.1)
Medical reasons	Health problems	80 (9.3)
	Inadequate fitness/stamina	13 (1.5)

### Logistic Regression

A total of seven factors emerged from the two regression analyses as significantly associated with canine success rates; dog breed, housing style, trial participation, age at acquisition, use of electric collars, hypothetical maximum treatment expenditure and owner conscientiousness score.

### Canine Factors

#### Dog Breed

The breed of the working dogs currently used by the respondents was significantly associated with success rates of recruited dogs. It can be seen in Table 3 that owners of a cattle dog crossbred reported below average success significantly more often than other dog breed owners and had the lowest mean probability of reporting average or above average success rates.

**Table 3:** Breeds of dogs owned by survey respondents who reported either below average success rates (<80%) or average and greater success rates (≥80%) of dogs acquired for stock work.

Variable	p-value (Wald test)	Mean probability of average or greater success	Standard error of the proportion	Number of responses
Dog Breed	**0.027**			
Border collie	Reference level	0.60	0.03	288
Border collie cross	0.434	0.52	0.04	127
Cattle dog	0.670	0.67	0.09	24
Cattle dog cross	0.013	0.30	0.10	20
Coolie	0.558	0.58	0.10	26
Coolie cross	0.788	0.63	0.11	19
Kelpie	0.050	0.66	0.01	1,078
Kelpie cross	0.179	0.64	0.04	151
Other	0.309	0.68	0.06	71

Summarised are the p-values emerging from stepwise backward elimination logistic regression analysis, the probability of average or greater success and the number of each breed reported.

#### Housing methods

The way in which respondents housed their stock herding dogs was associated with the cull rates they reported, exposing a significant difference between owners who housed their dogs on a chain and those who provided either an individual pen or a group pen for their working dogs. As indicated in Table 4, the highest probability of having average or greater success rates belongs to respondents housing their dogs in a group yard/pen while the respondents housing their working dogs in a group cage or on a chain have the lowest probability of having average or greater success rates.

**Table 4:** Housing style provided to dogs owned by survey respondents who report either below average success rates (<80%) or average and greater success rates (≥80%) of dogs acquired for stock work.

Variable	p-value (Wald test)	Mean probability of average or greater success	Standard error of the proportion	Number of responses
Housing of dogs	**<0.001**			
Individual shelter on chain	Reference level	0.56	0.02	584
Group shelter with yard/pen	<0.001	0.77	0.03	151
Group cage	0.855	0.53	0.09	30
Individual cage	0.111	0.62	0.02	488
Individual shelter with yard/pen	<0.001	0.71	0.02	457
Indoors with humans	0.094	0.65	0.05	96

P-values emerging from stepwise backward elimination logistic regression analysis, the probability of average or greater success and the number of dogs in each housing design are reported.

#### Working dog trial participation

As seen in Table 5, 267 of the 1,806 herding dogs described participated in working dog trials. Forty-three of these dogs were used only for trials. The remaining 84% performed herding work outside of competitions. Dogs competing in working dog trials had a significantly greater chance of falling into the group of respondents reporting average or above-average success rates.

**Table 5:** Total numbers of dogs participating and not participating in working dog trials that are owned by respondents reporting below average (<80%) and average or above average (≥80%) recruitment success rates.

Variable	p-value (Wald test)	Mean probability of average or greater success	Standard error of the proportion	Number of responses
Dog trial participation	**0.034**			
Yes	Reference level	0.70	0.03	267
No	0.034	0.62	0.01	1,539

P-values emerging from stepwise backward elimination logistic regression analysis and the probability of average or greater success are reported.

#### Age of dog at acquisition

Respondents who had acquired a dog when it was older than six months of age reported below average success rates significantly more often than those acquiring pups at a younger age or breeding their own working dogs. Table 6 details the probability of average or greater success for respondents according to the age they acquired dogs.

**Table 6:** The age dogs were acquired by owners who report either below average success rates (<80%) or average and greater success rates (≥80%) of dogs acquired for stock work.

Variable	p-value (Wald test)	Mean probability of average or greater success	Standard error of the proportion	Number of responses
Age dog was acquired	**0.002**			
Less than 8 weeks	Reference level	0.66	0.03	312
8–12 weeks	0.827	0.67	0.02	524
3–6 months	0.149	0.61	0.04	158
Older than 6 months	<0.001	0.56	0.03	318
Owner-bred	0.473	0.65	0.02	494

P-values emerging from stepwise backward elimination logistic regression analysis, the probability of average or greater success and the number of dogs in each age group are reported.

### Owner Factors

#### Use of electric shock collar (e-collar) in training

The vast majority of respondents (93%) do not use e-collars to train their working dogs. However, Table 7 indicates that below average success rates were reported significantly more often by respondents who do use e-collars.

**Table 7:** The number of respondents who report using electric shock collars in training and report either below average success rates (<80%) or average and greater success rates (≥80%) of dogs acquired for stock work.

Variable	p-value (Wald test)	Mean probability of success	Standard error of the mean	Number of responses
Use of electric collar in training	**0.001**			
No	Reference level	0.65	0.02	759
Yes	0.001	0.39	0.07	53

P-values emerging from stepwise backward elimination logistic regression analysis and the probability of average or greater success are reported.

#### Hypothetical maximum expenditure to save best working dog from illness or injury

**Figure 1:**
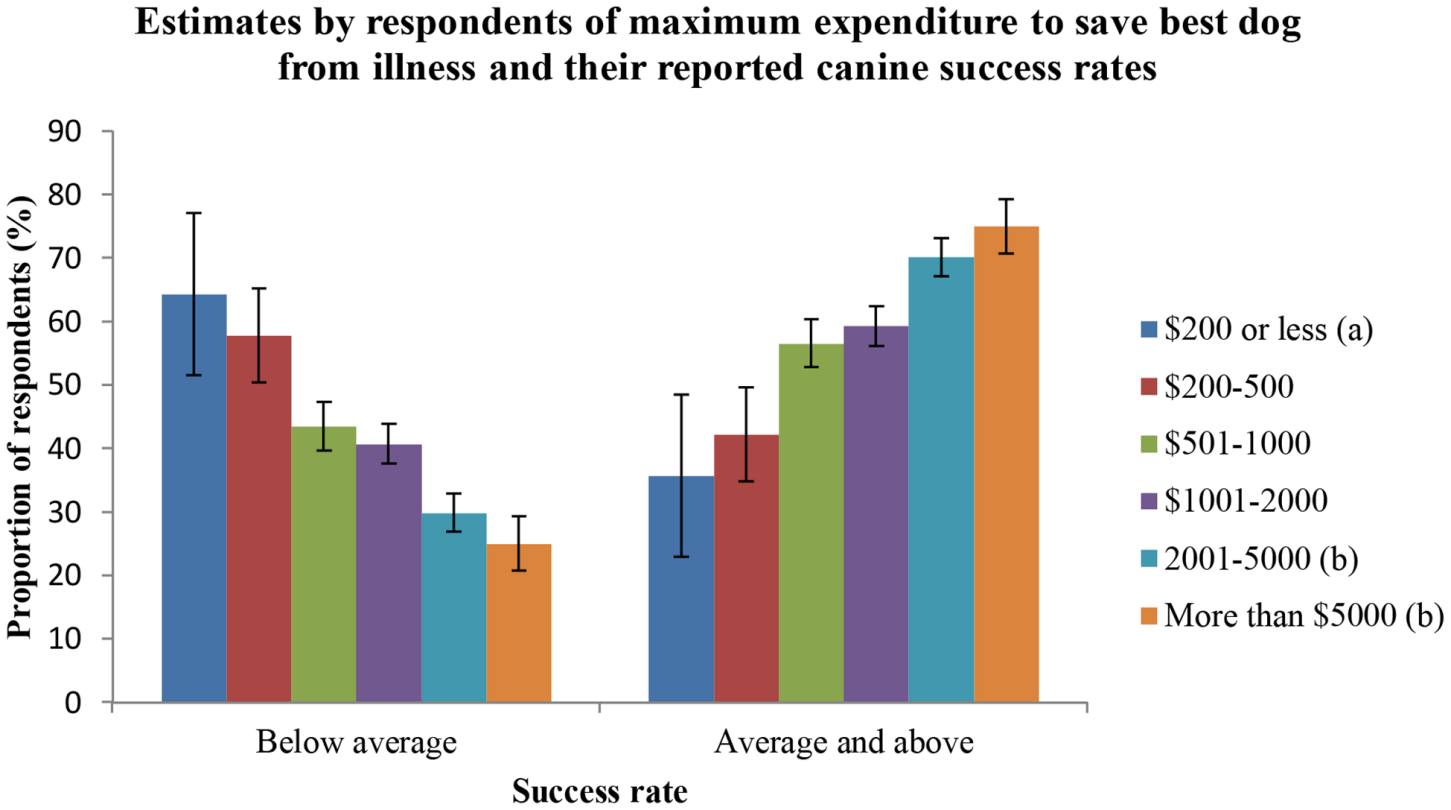
Success rates and respondents' estimates of maximum expenditure to save their best dog. A comparison of the proportion of respondents prepared to spend different amounts of money to save their best dog from a hypothetical illness or injury who report either below average success rates (<80%) or average and greater success rates (≥80%) of dogs they acquire for stock work. Error bars = ± SE(p%). a) reference level, b) significant difference from the reference level (p<0.05).

Figure 1 shows a positive association between the amounts of money respondents would be prepared to spend to treat their best dog to return it to work and the frequency with which they report success rates that are average and above (p<0.001).

#### Owner personality – conscientiousness score

**Figure 2:**
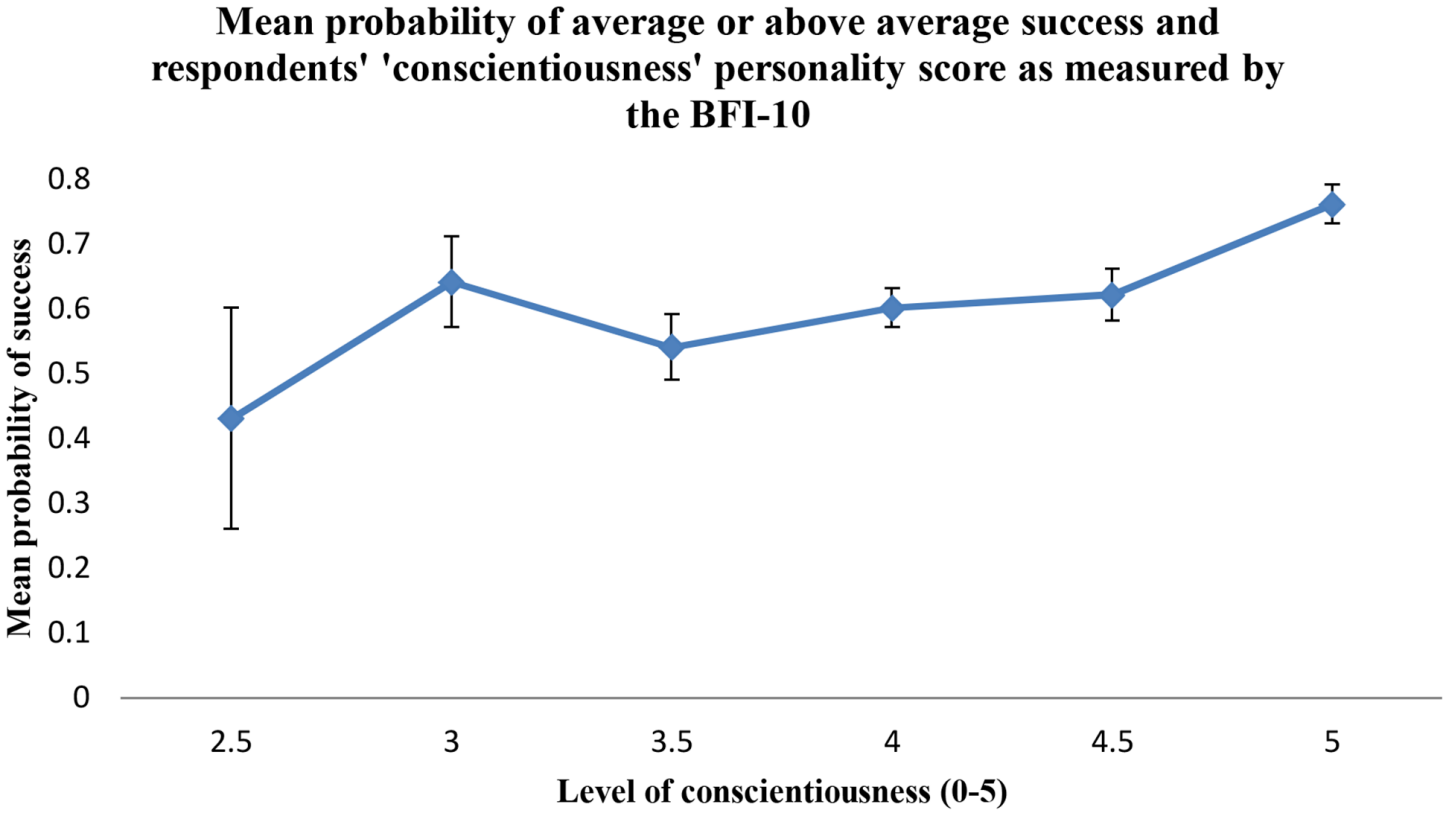
Probabilities of average or greater success and conscientiousness of handler. Mean probabilities of respondents with conscientiousness personality scores from two and a half to five (as measured by the BFI-10) reporting an average or greater success rate (≥80%) of the dogs they acquire for stock work. Error bars = ± SE(p).

Of the five personality traits tested, only conscientiousness was significantly associated with respondents' self-reported canine recruitment success rates (p = 0.007). All respondents scored between two and a half (n = 9, SE = 0.17, reference level) and five (n = 178, SE = 0.03) with a mode score of four (n = 281, SE = 0.03). As indicated in Figure 2, a trend was observed of increasing success rate with increasing conscientiousness score.

### Chi-Squared Analysis

Of the 26 variables dropped from the regression models, five were found to be significantly associated with success rate during chi-squared testing; training level at acquisition, insurance status, training with positive reinforcement, canine exercise frequency and the handler's view of their dog. Table 8 lists the variables dropped from the regression model that were not significant in chi-squared analysis.

**Table 8:** Variables dropped from the regression models that were not found to be significantly associated with respondents' success rates in chi-squared analysis.

Variable	p-value	X^2^-value (degrees of freedom)
**Canine Factors**		
Origin – external breeder or home-bred	0.47	1.51 (2)
Purchase price	0.10	10.7(6)
Sex	0.43	2.73 (3)
Veterinary costs in the last five years	0.79	1.06 (3)
Work type – utility, mustering, yard or trial only	0.09	6.54 (3)
**Owner Factors**		
Absence of dog training education (including books, training schools)	0.42	0.66 (1)
Attendance at dog training school(s)	0.44	0.60 (1)
Age	0.16	7.88 (5)
Agreeableness score	0.32	11.49 (10)
Breeder status	0.52	0.41 (1)
Certification in dog training	0.58	0.31 (1)
Extraversion score	0.16	11.74 (8)
Extent of inbreeding employed	0.65	2.48 (4)
Gender	0.50	0.45 (1)
Neuroticism score	0.38	8.59 (8)
Number of dogs owned	0.09	4.83 (2)

### Canine Factors

#### Training level at acquisition

**Figure 3:**
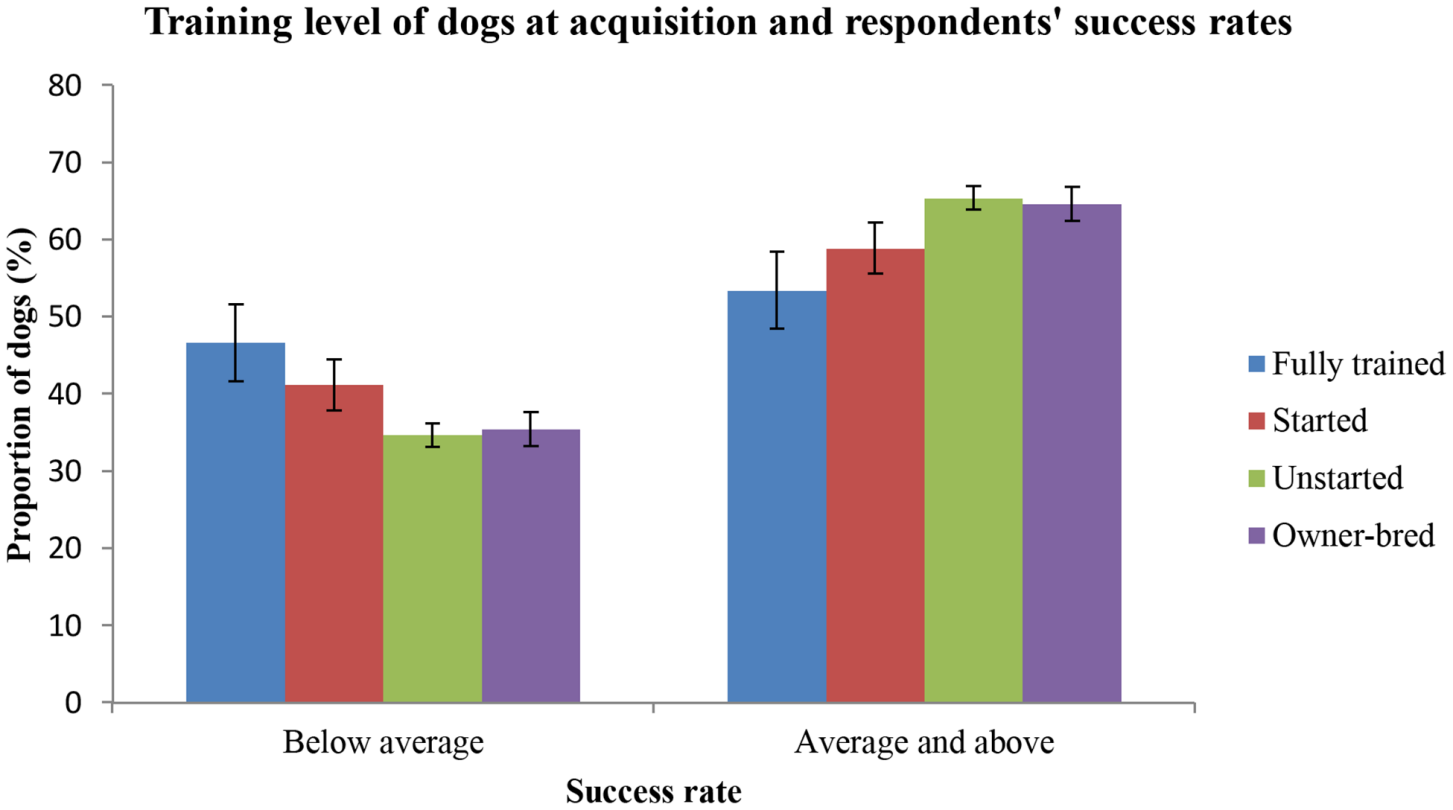
Training level of dogs at acquisition and respondents' success rates. Comparison of the proportion of dogs acquired with four different training levels by respondents who report either below average success rates (<80%) or average and greater success rates (≥80%) of dogs they acquire for stock work. Error bars = ± SE(p%).

The variable related to the prior training level of dogs upon acquisition by respondents was dropped from the regression model but showed a significant association with success rates when tested during chi-squared analysis (p = 0.03, X^2^ = 8.8 (3)). Figure 3 shows a trend of increasing probability of average or higher success rate as the extent of training at acquisition decreases. The majority of dogs were acquired unstarted (n = 967, SE = 0.02), 27% were bred by the current owner (n = 494, SE = 0.02), 13% were started at acquisition (n = 226, SE = 0.03) and the smallest group were the fully trained dogs (n = 118, SE 0.05).

#### Insurance status

Chi-squared analysis revealed a significant association between success rate and positive dog insurance status. However, Table 9 reveals that relatively few respondents report insuring their dogs.

**Table 9:** The insurance status of respondents' dogs.

Variable	p-value	X^2^-value (degrees of freedom)	Mean probability of average or greater success	Standard error of the proportion	Number of responses
Insurance status	**0.04**	4.1 (1)			
Insured			0.67	0.04	155
Not insured			0.63	0.01	1,651

Summarised are the p-value, X^2^ value, the mean probability of average and greater success (≥80%) and the total number of dogs in each insurance category.

### Owner Factors

#### Use of positive reinforcement in training

Although dropped from the regression model, the use of positive reinforcement in dog training was significantly associated with success rate in the chi-squared analysis (p = 0.01, X^2^ = 6.3(1)). Table 10 shows that the majority of respondents employ some form of reward for at least part of their training. This group of respondents had an increased probability of reporting average or greater success rates.

**Table 10:** The number of respondents using positive reinforcement during dog training.

Variable	p-value	X^2^-value (degrees of freedom)	Mean probability of average or greater success	Standard error of the proportion	Number of responses
Use of positive reinforcement in training	**0.01**	6.3 (1)			
No			0.55	0.11	22
Yes			0.63	0.02	790

Summarised are the p-value, X^2^ value and the mean probability of average and greater success (≥80%).

#### Exercise frequency

**Figure 4:**
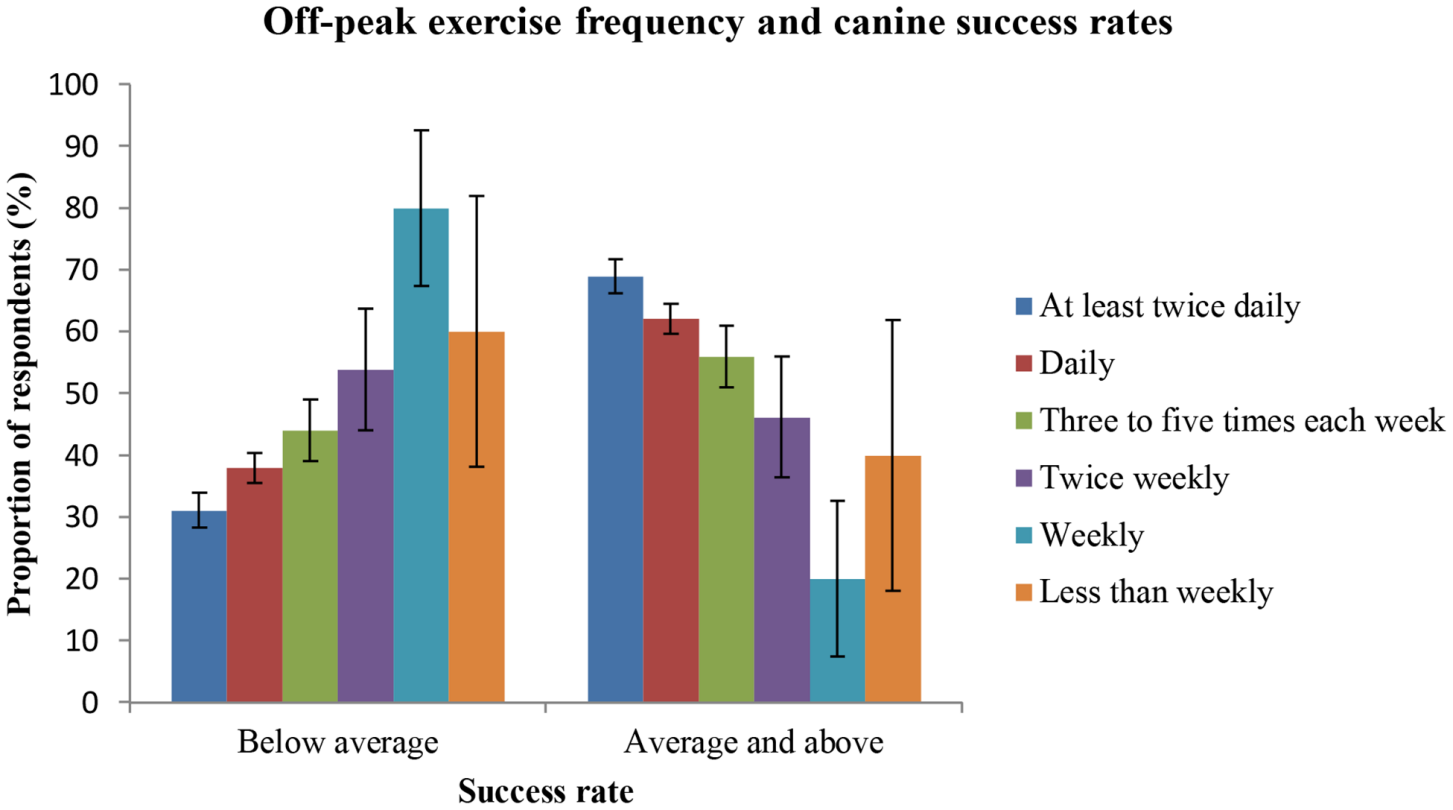
Frequency of exercise during off-peak periods and respondents' success rates. Comparison of the proportions of owners providing exercise to their dogs at six different frequencies, during off-peak farm work periods, who report either below average success rates (<80%) or average and greater success rates (≥80%) of dogs they acquire for stock work. Error bars = ± SE(p%).

Success rate was positively associated with exercise frequency (p = 0.003, X^2^ = 18(5)) as seen in Figure 4. The highest probability of reporting 80% success or more was 0.66 (n = 264, SE = 0.03) and applied to respondents who exercise their dogs at least twice daily. In contrast, the mean probability of greater success for those exercising their dogs less than weekly was 0.57 (n = 5, SE = 0.22).

#### Respondent's view of their working dogs

**Figure 5:**
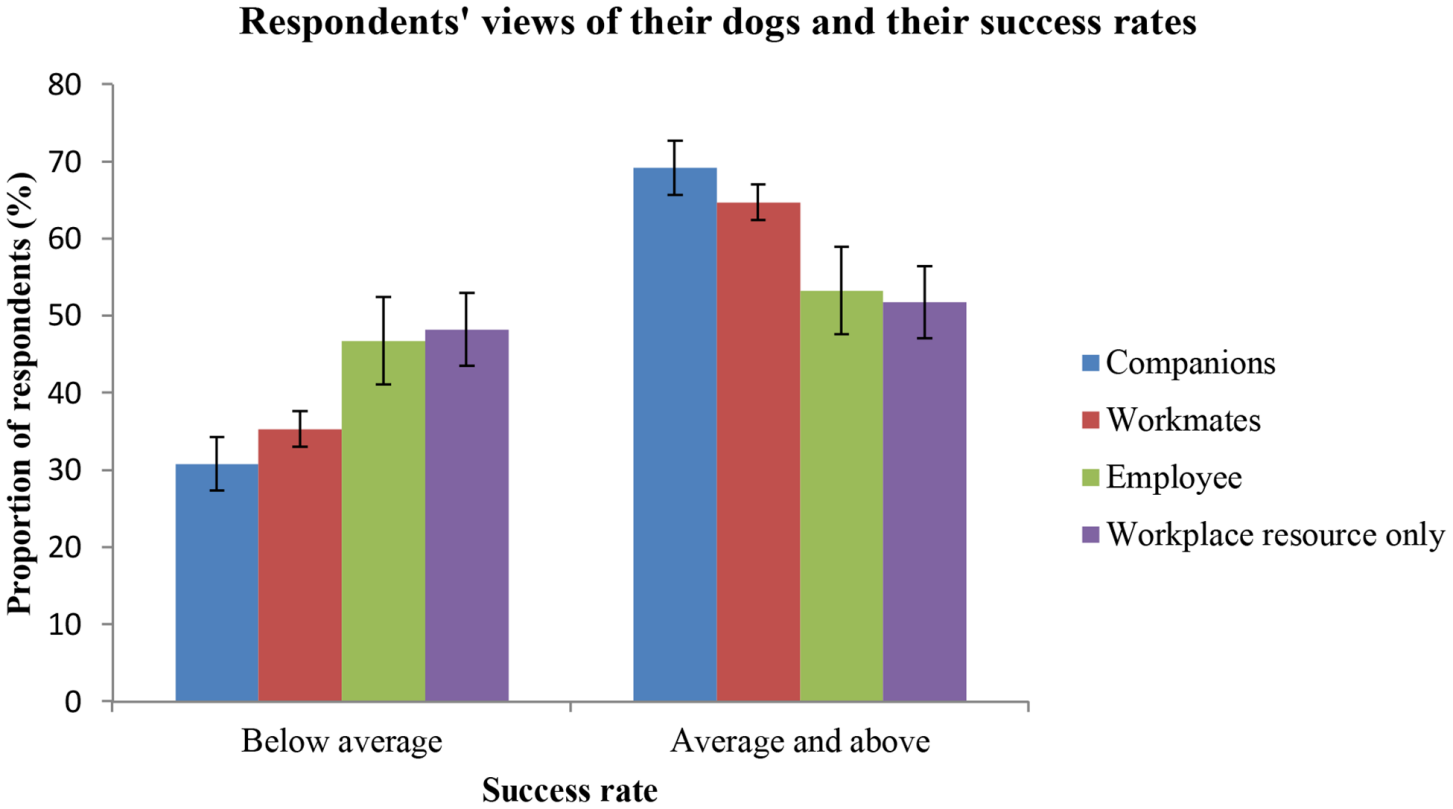
Respondents' views of their dogs and their success rates. Comparison of the proportions of respondents viewing their dogs in one of four ways who reported either below average success rates (<80%) or average and greater success rates (≥80%) of dogs they acquire for stock work. Error bars = ±SE(p%).

In chi-squared testing a significant association was found between the view handlers took of their dogs and their reported recruitment success rates (p = 0.006, X^2^ = 12.4(3)). Fifty-four per cent of respondents viewed their dogs as “workmates”. These respondents had the same probability of average or greater success (p = 0.63, n = 436, SE = 0.02) as respondents who viewed their dogs as “employees” (p = 0.63, n = 77, SE = 0.06). A probability of average or greater success of 0.65 (n = 172, SE = 0.04) belonged to the 21% of respondents who viewed their dogs as “companions”. Figure 5 shows that respondents who viewed their dogs as “a workplace resource only” recorded the highest proportion of below average success. These respondents has a probability of average or above success of 0.59 (n = 114, SE = 0.05).

## Discussion

Working dog success rates may be definitively measured by several of the organisations raising and training guide dogs [12], [25], [26], detection dogs [27], [28] and military dogs [13]. These facilities can keep records of the total number of dogs purchased or bred for training, follow a testing protocol to assess the progress of these dogs and elect to pass or fail them based on the results. Although the validity of these protocols is rarely established [29], they permit quantification of recruitment success beyond what is currently possible for stock herding dogs. The current results address some of that shortfall and provide the greatest insight, to date, into the success rates of Australian herding dogs and the reasons given for their failure.

The reasons for culling working dogs are overwhelming described as behavioural in nature in the current, and in previous, studies [10], [13]. To address this, several authors have examined the heritability of behavioural traits valued in working dogs [30]–[33]. Heritability estimates for behavioural traits, although often low to moderate, are usually sufficiently high for genetic gain to be possible through selective breeding. Low heritability estimates may result from imprecise behavioural evaluations but also emphasise that the environment's role in the manifested behaviours is significant. The current study identifies non-genetic factors with a possible effect on stock working dog success rates.

The nature of survey data requires a consideration of their accuracy. For example, respondents were asked to make estimations of their culling history which are undoubtedly subject to recall bias. High cull rates and certain management practices are at risk of being under-reported due to the human tendency towards “Socially Desirable Responding” [34]. The assurance of anonymity given to survey respondents was intended to at least partially mitigate this outcome. An additional limitation of the study, which must be acknowledged, is the recruitment method used to enlist participants. A random sample of herding dog owners could not be assured. Despite this, the sample achieved was representative of the Australian farming population in several aspects. For a comparison of the characteristics of the survey participants with those of the Australian farming population see Arnott et al. [11]. This study does not imply causation between the variables investigated and working dog cull rates. The results serve to document common working dog management practices on farms and the associations serve to direct further research into areas of potential benefit to the dog-handler dyad.

A working dog is at risk of underperforming if good health and welfare are not adequately maintained. A link between compromised welfare and performance has been well demonstrated as frustrated, apathetic or fearful animals have difficulty learning and concentrating [15], [35]–[37]. Welfare may be compromised by failing to meet a dog's needs for socialisation [38], stimulation [39], [40] and comfort [37] or by causing it fear [41] or pain [42]. However, the exact “needs” of the dog are yet to be definitively established [43]. Identifying management practices that were associated with below average success in herding dogs, leads one to consider the impact the practices may have on canine welfare and, subsequently, on learning and performance.

Confinement of dogs, and other animals, can result in behavioural abnormalities [44], [45]. Although optimal housing for working dogs has not been established, the influence of housing on performance is likely to be significant. Beerda et al. [45] investigated the effects of social and spatially restricted housing on dogs. When challenged by novel or startling stimuli, dogs that had been chronically stressed by restricted housing conditions, displayed behaviours considered indicative of uncertainty, aggression and excitement. These behaviours can all be reasons for poor performance and even dismissal in herding dogs [46], [47].

This study documented the canine housing methods employed by Australian farmers. It was assumed that, regardless of housing style, some form of shelter was supplied to the dogs, in accordance with current codes of practice [23]. No information was gathered about the size of enclosures, the lengths of tethers or the numbers of dogs combined in group housing. Most of the dogs reported in this study were kept on chains. The prevalence of chaining, as a method of housing working dogs, can be explained by the low cost involved as minimal infrastructure is required. However, the greatest probability of achieving higher success rates was among respondents who housed their dogs in group pens or yards. There are several benefits to this style of kennelling which could confer an advantage to the dogs. Firstly, freedom from tethering or caging gives the dogs the ability to exercise more choice and control within the environment. Secondly, the dogs are provided with the enrichment of conspecific socialisation, including play. Multiple studies report an increased risk of repetitive behaviours in individually housed dogs which are believed to indicate compromised psychological wellbeing [38], [48]. Hetts et al. [49] showed that, compared to dogs housed in social isolation, dogs housed in pairs vocalised less, had fewer stereotypies and longer sleep duration. However, in the current study, owners who housed their dogs in group cages did not report success rates that were significantly different to owners who chained dogs (p = 0.53). It is possible that this could reflect an issue with stocking density or enclosure size, but the very small sample size (n = 30 for group cages) makes it unwise to draw conclusions. It appears that the group pen environment benefits welfare by ameliorating some of the frustration which can arise from kennelling and may result in a well-rested, well-adjusted working dog capable of reaching its potential.

Respondents who kept a dog individually in a yard or pen also had a significantly higher probability of average or greater success than those respondents chaining their dogs (p<0.001). A significant difference was not revealed for individually caged dogs (p = 0.11). Data were not collected to establish if the pens and yards provided dogs with advantages of size, hygiene, environmental complexity or perceived freedom of movement compared to cages and tethering. As previous studies have often focused on laboratory, shelter and military dogs [48]–[50], tethering has not closely been examined. A study of sled dogs comparing tethering to penning described some differences in the behaviour of the dogs in the two environments. However, conclusions could not be drawn on the relevance of these differences to the welfare of the dogs [51].

A small proportion of the dogs described (5%) were kept inside the house with the handler. Although the probability of these respondents reporting higher success rates was greater than those who chained their dogs, this difference failed to reach significance (p = 0.094). Although the standard error suggests a disparity between the sample and true population means, it must be considered that there may be a real tendency for decreasing success rates among dogs kept in the home when compared to those kept in outside group or individual shelters. Although the home environment is expected to meet the dog's needs in terms of comfort and human socialisation, some professional dog trainers suggest a training-related disadvantage associated with this arrangement. When trained responses are not required, out of work periods, isolating the dog from the handler reduces generalisation (animals giving trained responses to cues similar to those used in training) [52].

Logically, the importance of the style of housing provided to the dogs diminishes with the time they are required to be housed. Stock herding dogs have periods of frequent and intense work during peak activity on farms [11] but, for most dogs, this activity level is not maintained all year. During off-peak periods on farms, where stock handling tasks are at a minimum, the dogs are at risk of long periods of kennelling. The significance of these off-peak periods is suggested by the finding that the number of hours worked during peak periods on farm was not significantly associated with reported success rates (p = 0.11, X^2^ = 6.11(3)). However, the frequency of exercise provided to the dogs during off-peak periods was significantly associated with success rate (p = 0.003, X^2^ = 18.0(5)). A positive correlation existed between the probability of having average or above average success rates and increasing frequency of exercise (from once weekly to at least twice daily).

The benefits of keeping the dogs physically fit and conditioned for work are clear especially when considering the requirement for some to travel up to 30 km per day while working [53]. Working dog enthusiasts frequently refer to the need for their dogs to display “stamina”, “heart” and “endurance” [46], [47] which can only be expected of a dog who is allowed to maintain its fitness [54]. However, respondents were only asked the frequency, and not the nature, of the exercise. The questionnaire described exercise as “time spent off the chain or out of confinement”. This would include any time interacting with the outside environment or handler and is not limited to aerobically demanding activity. Therefore, the benefits of “exercising” the working dog should be considered greater than merely physical conditioning.

Lefebvre (2009) reported that military working dogs had lower cortisol concentrations when receiving exercise and human contact twice daily compared to twice weekly. It was hypothesised that the cortisol elevations in the latter group of dogs indicated compromised welfare due to more prolonged periods of social isolation and a subsequent effect on the dog-handler bond. These issues may be pertinent to the success of herding dogs that are not given regular stimulation and positive interaction with their owners. The result may be a dog that under-performs and is therefore at an increased risk of being culled.

The importance of enrichment and human interaction are also suggested by the positive correlation between success rates and participation in working dog trials. It could be argued that the increased probability of average or above success rates for respondents competing in trials is associated with the work that is required of their dogs. There may be a perception that trial dogs require a less extensive skill set than dogs engaged in farm work and, as a result, they are subject to less selection pressure and fewer culls. However, 84% of the dogs competing in trials are also farm working dogs requiring that they are able to perform both roles adequately. In addition, there was no significant association between success rates and the types of work performed by the respondents' dogs - mustering, yard, utility and trial only (p = 0.09, X^2^ = 6.5(3)). A second hypothesis is that the benefits of trialling arise from being owned by handlers with greater training knowledge or experience. However, while there was a significant association between trial participation and attendance at dog training schools (p<0.001, unpublished data), there is no such relationship between the attendance at training schools and canine success rates (p = 0.44, X^2^ = 0.6(1)). This is consistent with the findings from previous studies that trainer experience did not appear to be a major determinant of working dog performance and obedience in search dogs [16] and military dogs [39]. That said, Lefebvre et al (2007) identified a welfare and performance benefit in Belgian military working dogs who engaged with their handlers in sporting activities off duty. The authors suggest that the performance benefits may arise from an improved handler-dog bond. Therefore, the increased success rates associated with trial participation by farm dogs could be related to the additional time handlers spend with the dogs to prepare for, and participate in, competitions.

The respondents' perceptions of their dogs as companions, workmates, employees or solely workplace resources were associated with success rates. The dog-handler bond, again, emerges as a possible influence on the effectiveness of the working dog. The relationships between working dogs and their handlers are arguably more complex than those that arise in a companion animal setting not least because they must operate at a distance from one another. This complexity also reflects an inherent conflict that exists for working dog handlers in “the definition and treatment of animals as functional objects, on one hand, and sentient individuals, on the other” [18]. That working dogs exist to perform a function with economic implications bears a similarity to other production animals. Wilkie [55] examined the issue of farmers' attitudes to these “sentient commodities”. She proposed a model to describe the human-livestock interaction defined by the quadrants ‘detached detachment’, ‘concerned detachment’, ‘concerned attachment’ and ‘attached attachment’. These states were defined by certain perceptions of the animal which may alter for an individual over time and circumstance. Each shares a commonality, respectively, with the descriptors ‘workplace resource only’, ‘employee’, ‘workmate’ and ‘companion’. Wilkie [55] asserts that how farmers perceive and relate to livestock goes to the core of the practice of ‘stockmanship’. Equally, how farmers perceive their working dogs is likely to have implications for the quality of their ‘dogmanship’. Whether they form a social relationship with, or remain detached from, their dog may affect the training and care of the animal. Additionally, the performance and perceived potential of the dog may affect the handler's relationship with the dog. The results from this study show a positive correlation between the degree of “attachment” by the owner to their dogs and the probability of average or greater success rates. Whether respondents who have an attachment to their dogs produce fewer underperforming dogs, or are less willing to cull underperforming dogs, is unclear from the current data.

Somewhat surprisingly, owners who acquired dogs that were considered fully trained, had the highest probability of reporting lower than average recruitment success rates. This was also the finding for respondents acquiring dogs over six months of age. The highest probability of being in the greater success group belonged to those respondents who acquired unstarted dogs and dogs less than six months of age or bred their own dogs. This may be another example of the importance of the dog-handler bond as, by necessity, more time must be spent with an untrained animal to reach a point of competency. Farmers may also opt to purchase an older, fully trained dog if they feel they do not have a lot of time to spend with a dog. There is also the possibility that older, fully trained dogs are purchased by farmers who do not have the confidence, knowledge or aptitude to effectively handle and train dogs which may compromise their success rates. Finally, it must also be considered that a breeder willing to sell a dog older than six months has identified it as an animal not worthy of keeping as breeding stock or for their own purposes.

The nature of the time spent with the working dogs and the methodology of training do appear, at least by association, to be relevant to the success outcomes of the dogs. Use of positive reinforcement in training was significantly associated with average and above success rates. The effect of training method on obedience has also been investigated in companion dogs. Hiby et al. [14] employed an owner questionnaire to identify training outcomes. The owners' assessments of their dogs' obedience were positively correlated with the use of rewards in training. When specific training tasks were examined, such as heeling or giving up an object, reward frequency was associated with higher levels of obedience.

To be categorised as using positive reinforcement in training, Farm Dog Survey respondents were not required to do so exclusively. Many described also using training methods involving negative reinforcement, negative punishment or positive punishment. Seven per cent of respondents (n = 53) reported using electric collars in training. Twenty-one of these lived in states of Australia where the use of the device is prohibited. The prevalence of e-collar use reported by Farm Dog Survey respondents is double the estimated prevalence for the British general dog-owning population [56]. This may reflect a different attitude to training working dogs compared to companion dogs. In the current study, training with an electric shock collar was significantly associated with reports of below average success rates (p = 0.001). There is considerable evidence that aversive stimuli used in dog training can be detrimental to performance and welfare [15], [17], [56]–[58]. In a comparison of e-collars and positive reinforcement used in training, significantly fewer owners using e-collars reported success [56]. When an e-collar was compared to a pinch collar and a pre-trained “quitting signal” in an obedience exercise, the dogs trained with an e-collar learned the fastest [42]. However, the study design resulted in the e-collar stimulus being administered with the correct timing more reliably than the other two training cues. As noted by the authors, this factor may have given the e-collar an advantage as, regardless of the training method used, consistency and timing are crucial to effective training [57], [59].

The current study cannot confirm that the use of e-collars causes dog training failure. The respondents may be resorting to aversive training techniques when experiencing performance problems with their dogs arising from other factors. Nevertheless, the results do suggest that e-collars are not providing a solution to the performance problems.

The probability of a respondent reporting average or greater success rates increased with the amount of money they estimated that they would be prepared to pay to treat their best working dog to allow it to return to work. In the Farm Dog Survey, successful dogs lost to illness or injury were considered “retired” and were not included in the cull rates. Therefore, this association reflects an attitude of the owner to their dogs that influences other areas of their management that may ultimately affect their success rates. Owners prepared to invest substantially in the treatment of their dog may appreciate the value of the dog to their farming enterprise which has been estimated to be approximately $40 000 over the dog's working lifetime [11]. Similarly, respondents who insured their working dogs reported significantly lower cull rates. The decision to insure the dogs also indicates recognition of the value of the dogs. This recognition may translate into a preparedness to dedicate more time and resources into their working dogs and, in doing so, increase the chances of the dogs working successfully. Purchase price of the dogs failed to reach significance when tested for an association with recruitment success rates. However, respondents who had purchased the most expensive dogs did report average or greater success rates more often than other respondents.

The breed of dog respondents employed was associated with reported success rates (p = 0.027). Cattle dog crossbred dogs were the only breed group differing significantly to the reference breed (p = 0.013). Cattle dogs are frequently selected for a forceful style of work which can include nipping or biting livestock. These behaviours could be inappropriate if used in the wrong context and may be difficult to control for a handler. However, purebred cattle dogs had one of the higher probabilities of being associated with average or above success (0.67) so it is difficult to explain why the cattle dog crossbreds had a comparatively low probability of success (0.30). No such disparity existed between other purebred and crossbreed groups and no further details were gathered on the parentage of the crossbreds. The small sample size of cattle dogs and cattle dog crosses may have affected the results.

Efforts to improve working dog success rates have focused largely on improving the suitability of the dogs particularly with respect to their temperament and genetic predispositions. However, there is a growing interest in the role that the owner or handler personality has in influencing a dog's potential. Kis et al. [60] found that owner personality (as measured by the BFI) did appear to play a role in their dog's performance and behaviour during a simple interaction task. The current respondents showed a positive relationship between increasing conscientiousness scores and increased probability of belonging to the higher canine recruitment success group. None of the other four personality dimensions were significantly associated with success rates. In the human psychology literature, conscientiousness is frequently associated with positive outcomes for people [61], [62]. Barrick and Mount [61] examined the Big Five personality traits for their relationship with job performance in people across five occupational groups. Conscientiousness was consistently associated with success in all job performance criteria across all occupational categories. The trait encompasses characteristics of perseverance, organisational ability, ambitiousness and self-discipline [63]. These attributes may lead a handler to work harder to make a dog a success. It is also worth considering that an aspect of this human personality trait is inherently effective in communicating with dogs. For example, consistent behaviour has been attributed to the conscientiousness trait [64], [65]. As previously discussed, consistency plays an important role in effectively communicating with animals. Confusion and distress result if it is impossible for an animal to reliably predict the outcome of their actions [35]. Similarly, Arhant et al. (2010) demonstrated correlations between owner inconsistency and disobedience, fear and anxiety in their dogs. Therefore, the typical behaviour of a working dog handler with a conscientious personality may foster the desirable traits of obedience and emotional stability in their dogs.

## Conclusions

The current study shows that a number of husbandry practices and human traits are associated with canine outcomes. The significance of housing, exercise frequency and training technique suggests the importance of addressing canine welfare standards. Factors such as handler personality, view of their dogs, involvement in dog trials and the training level of the dogs when acquired infer a need to foster the canine-human bond to optimise success. These findings demand recognition of the role the dog handler has in influencing results. They should help avoid the animals being charged with the sole responsibility for success or failure. The study findings provide a guide for areas of further investigation for optimising care and management of Australian stock herding dogs. The insights also have potential relevance to companion dogs and other working dog sectors. Future research will be crucial in providing robust evidence for working dog codes of practice rather than relying on recommendations for arbitrary or purely cosmetic change.

## Supporting Information

Questionnaire S1 **Farm Dog Survey.**
(PDF)

## References

[1] Keating BA, Carberry PS (2010) Emerging opportunities and challenges for Australian broadacre agriculture. Crop & Pasture Science 61: 269–278.

[2] Allan MF, Smith TPL (2008) Present and future applications of DNA technologies to improve beef production. Meat Science 80: 79–85. 22063172 10.1016/j.meatsci.2008.05.023

[3] Bakker DM, Hamilton GJ, Houlbrooke DJ, Spann C (2005) The effect of raised beds on soil structure, waterlogging, and productivity on duplex soils in Western Australia. Australian Journal of Soil Research 43: 575–585.

[4] Polkinghorne RJ, Thompson JM (2010) Meat standards and grading A world view. Meat Science 86: 227–235. 20541325 10.1016/j.meatsci.2010.05.010

[5] Broderick S, Wright V, Kristiansen P (2011) Cross-case analysis of producer-driven marketing channels in Australia. British Food Journal 113: 1217–1228.

[6] Morales LE, Griffith G, Wright V, Fleming E, Umberger W, et al (2013) Variables affecting the propensity to buy branded beef among groups of Australian beef buyers. Meat Science 94: 239–246. 23501257 10.1016/j.meatsci.2013.02.005

[7] McNair Ingenuity Research Pty Ltd. (2012) Quantitative Agricultural Readership Survey.

[8] Australian Bureau of Statistics (2012) Australian Social Trends, Dec 2012. ABS, Canberra.

[9] Arnott ER, Early JB, Wade CM, McGreevy PD (2014) Estimating the economic value of Australian stock herding dogs. Animal Welfare 23: 189–197.

[10] Branson N, Cobb M, McGreevy P (2009) Australian Working Dog Survey Report. Canberra, ACT: Department of Agriculture Fisheries and Forestries, Australian Animal Welfare Strategy.

[11] Arnott ER, Early JB, Wade C, McGreevy PD (2014) Estimating the economic value of Australian stock herding dogs. Animal Welfare In press.

[12] Goddard ME, Beilharz RG (1982) Genetic and environmental factors affecting the suitability of dogs as guide dogs for the blind. Theoretical and Applied Genetics 62: 97–102. 24270555 10.1007/BF00293339

[13] Evans RI, Herbold JR, Bradshaw BS, Moore GE (2007) Causes for discharge of military working dogs from service: 268 cases (2000–2004). Journal of the American Veterinary Medical Association 231: 1215–1220. 17937551 10.2460/javma.231.8.1215

[14] Hiby EF, Rooney NJ, Bradshaw JWS (2004) Dog training methods: their use, effectiveness and interaction with behaviour and welfare. Animal Welfare 13: 63–69.

[15] Haverbeke A, Laporte B, Depiereux E, Giffroy JM, Diederich C (2008) Training methods of military dog handlers and their effects on the team's performances. Applied Animal Behaviour Science 113: 110–122.

[16] Alexander MB, Friend T, Haug L (2011) Obedience training effects on search dog performance. Applied Animal Behaviour Science 132: 152–159.

[17] Rooney NJ, Cowan S (2011) Training methods and owner-dog interactions: Links with dog behaviour and learning ability. Applied Animal Behaviour Science 132: 169–177.

[18] Sanders CR (2006) “The dog you deserve” - Ambivalence in the K-9 officer/patrol dog relationship. Journal of Contemporary Ethnography 35: 148–172.

[19] Ikerd JE (1996) Sustaining the profitability of agriculture. The economist's role in the agricultural sustainability paradigm. San Antonio, TX.

[20] Phillips CJC (2005) Ethical perspectives of the Australian live export trade. Australian Veterinary Journal 83: 558–562. 16164147 10.1111/j.1751-0813.2005.tb13336.x

[21] Wells AED, Sneddon J, Lee JA, Blache D (2011) Farmer's Response to Societal Concerns About Farm Animal Welfare: The Case of Mulesing. Journal of Agricultural & Environmental Ethics 24: 645–658.

[22] Barnett JL, Newman EA (1997) Review of welfare research in the laying hen and the research and management implications for the Australian egg industry. Australian Journal of Agricultural Research 48: 385–402.

[23] Victorian Government Department of Environment and Primary Industries (2013) The Code of Practice for the Operation of Breeding and Rearing Businesses. In: Industries DoEaP, editor. Melbourne: Victorian Government.

[24] Rammstedt B, John OP (2007) Measuring personality in one minute or less: A 10-item short version of the Big Five Inventory in English and German. Journal of Research in Personality 41: 203–212.

[25] Batt LS, Batt MS, Baguley JA, McGreevy PD (2008) Factors associated with success in guide dog training. Journal of Veterinary Behavior: Clinical Applications and Research 3: 143–151.

[26] Guide Dogs Queensland (2008) Withdrawn Guide Dogs.

[27] Maejima M, Inoue-Murayama M, Tonosaki K, Matsuura N, Kato S, et al (2007) Traits and genotypes may predict the successful training of drug detection dogs. Applied Animal Behaviour Science 107: 287–298.

[28] Rooney NJ, Gaines SA, Bradshaw JWS, Penman S (2007) Validation of a method for assessing the ability of trainee specialist search dogs. Applied Animal Behaviour Science 103: 90–104.

[29] Overall KL, Juarbe-Diaz S, Dunham AE, Branson N (2012) What do we really know about working dogs? A critical review of the literature.

[30] Schmutz SM, Schmutz JK (1998) Heritability estimates of behaviors associated with hunting in dogs. Journal of Heredity 89: 233–237. 9656465 10.1093/jhered/89.3.233

[31] Courreau JF, Langlois B (2005) Genetic parameters and environmental effects which characterise the defence ability of the Belgian shepherd dog. Applied Animal Behaviour Science 91: 233–245.

[32] Arvelius P, Malm S, Svartberg K, Strandberg E (2012) Measuring Herding Behavior in Border Collie - Effect of Protocol Structure on Usefulness for Selection. Journal of Veterinary Behavior: Clinical Applications and Research

[33] Arvelius P, Klemetsdal G (2013) How Swedish breeders can substantially increase the genetic gain for the English Setter's hunting traits. Journal of Animal Breeding and Genetics 130: 142–153. 23496015 10.1111/jbg.12026

[34] Paulhus DL (2002) Socially Desirable Responding: The Evolution of a Construct. In: Braun HI, Jackson DN, editors. The Role of Constructs in Psychological and Educational Measurement. Mahwah New Jersey: Erlbaum. pp. 49–69.

[35] Seligman ME (1972) Learned helplessness. Annual review of medicine 23: 407–412. 10.1146/annurev.me.23.020172.002203 4566487

[36] Vincent IC, Leahy RA (1997) Real-time non-invasive measurement of heart rate in working dogs: a technique with potential applications in the objective assessment of welfare problems. Veterinary Journal 153: 179–184. 10.1016/s1090-0233(97)80038-x 12463403

[37] Rooney N, Gaines S, Hiby E (2009) A practitioner's guide to working dog welfare. Journal of Veterinary Behavior-Clinical Applications and Research 4: 127–134.

[38] Taylor KD, Mills DS (2007) The effect of the kennel environment on canine welfare: a critical review of experimental studies. Animal Welfare 16: 435–447.

[39] Lefebvre D, Diederich C, Delcourt M, Giffroy JM (2007) The quality of the relation between handler and military dogs influences efficiency and welfare of dogs. Applied Animal Behaviour Science 104: 49–60.

[40] Lefebvre D, Giffroy J-M, Diederich C (2009) Cortisol and behavioral responses to enrichment in military working dogs. Journal of Ethology 27: 255–265.

[41] Horvath Z, Doka A, Miklosi A (2008) Affiliative and disciplinary behavior of human handlers during play with their dog affects cortisol concentrations in opposite directions. Hormones and Behavior 54: 107–114. 18353328 10.1016/j.yhbeh.2008.02.002

[42] Salgirli Y, Schalke E, Boehm I, Hackbarth H (2012) Comparison of learning effects and stress between 3 different training methods (electronic training collar, pinch collar and quitting signal) in Belgian Malinois Police Dogs. Revue De Medecine Veterinaire 163: 530–535.

[43] Stafford K (2012) Canine welfare: We know everything, don't we? Veterinary Journal 192: 257–257. 10.1016/j.tvjl.2012.02.007 22445766

[44] Fox MW (1965) Environmental factors influencing stereotyped and allelomimetic behavior in animals. Laboratory Animal Care 15: 363–&. 4222103

[45] Beerda B, Schilder MBH, Van Hooff J, De Vries HW, Mol JA (1999) Chronic stress in dogs subjected to social and spatial restriction. I. Behavioral responses. Physiology & Behavior 66: 233–242. 10336149 10.1016/s0031-9384(98)00289-3

[46] Gedye J ([2000]) Training notes for farmers re:Kelpies. [Ballarat].

[47] Parsons AD (2010) The Kelpie. Camberwell, VIC, Australia: Penguin Group, Australia.

[48] Hubrecht RC, Serpell JA, Poole TB (1992) Correlates of pen size and housing conditions on the behavior of kenneled dogs. Applied Animal Behaviour Science 34: 365–383.

[49] Hetts S, Clark JD, Calpin JP, Arnold CE, Mateo JM (1992) Influence of housing conditions on beagle behavior. Applied Animal Behaviour Science 34: 137–155.

[50] Gaines SA, Rooney NJ, Bradshaw JWS (2008) The Effect of Feeding Enrichment upon Reported Working Ability and Behavior of Kenneled Working Dogs. Journal of Forensic Sciences 53: 1400–1404. 18808374 10.1111/j.1556-4029.2008.00879.x

[51] Yeon SC, Golden G, Sung W, Erb HN, J RA, et al (2001) A Comparison of Tethering and Pen Confinement of Dogs. Journal of Applied Animal Welfare Science 4: 257–270.

[52] McGreevy P (2009) A modern dog's life. Sydney: UNSW Press.

[53] Hampson BA, McGowan CM (2007) Physiological responses of the Australian cattle dog to mustering exercise. Equine and Comparative Exercise Physiology 4: 37–41.

[54] Love SB, Davis MS, Goad C, Murphy K, Aichele DB, et al (2010) Predictive haematological and serum biomarkers for canine endurance exercise. Comparative Exercise Physiology 7: 109–115.

[55] Wilkie R (2005) Sentient commodities and productive paradoxes: the ambiguous nature of human-livestock relations in Northeast Scotland. Journal of Rural Studies 21: 213–230.

[56] Blackwell EJ, Bolster C, Richards G, Loftus BA, Casey RA (2012) The use of electronic collars for training domestic dogs: estimated prevalence, reasons and risk factors for use, and owner perceived success as compared to other training methods. Bmc Veterinary Research 8. 10.1186/1746-6148-8-93 PMC3474565 22748195

[57] Arhant C, Bubna-Littitz H, Bartels A, Futschik A, Troxler J (2010) Behaviour of smaller and larger dogs: Effects of training methods, inconsistency of owner behaviour and level of engagement in activities with the dog. Applied Animal Behaviour Science 123: 131–142.

[58] Blackwell EJ, Twells C, Seawright A, Casey RA (2008) The relationship between training methods and the occurrence of behavior problems, as reported by owners, in a population of domestic dogs. Journal of Veterinary Behavior-Clinical Applications and Research 3: 207–217.

[59] McGreevy P, Boakes R (2005) Carrots and sticks: principles of animal training. Cambridge: Cambridge University Press.

[60] Kis A, Turcsan B, Miklosi A, Gacsi M (2012) The effect of the owner's personality on the behaviour of owner-dog dyads. Interaction Studies 13: 373–385.

[61] Barrick MR, Mount MK (1991) The big 5 personality dimensions and job-performance - a metaanalysis. Personnel Psychology 44: 1–26.

[62] Shiner RL, Masten AS (2012) Childhood personality as a harbinger of competence and resilience in adulthood. Development and Psychopathology 24: 507–528. 22559127 10.1017/S0954579412000120

[63] Costa PT, McCrae RR (1992) The 5-factor model of personality and its relevance to personality-disorders. Journal of Personality Disorders 6: 343–359.

[64] Hofstee WKB, Deraad B, Goldberg LR (1992) Integration of the big-5 and circumplex approaches to trait structure. Journal of Personality and Social Psychology 63: 146–163. 1494982 10.1037//0022-3514.63.1.146

[65] Saucier G, Ostendorf F (1999) Hierarchical subcomponents of the Big Five personality factors: A cross-language replication. Journal of Personality and Social Psychology 76: 613–627. 10234848 10.1037//0022-3514.76.4.613

