# Supplementary material for: Environmental Factors Associated with Success Rates of Australian Stock Herding Dogs
Source: PLoS One. 2014 Aug 19;9(8):e104457. doi: 10.1371/journal.pone.0104457 (PMC4138039; doi:10.1371/journal.pone.0104457)
Supplement: Questionnaire S1 — Farm Dog Survey. (PDF) [file pone.0104457.s001.pdf]

Farm Dog Survey

About this survey

This survey is confidential. It should take about 15 minutes to complete. It is important that all questions are answered. The aim of this survey is to investigate the use of stock herding dogs in Australia.

By completing this survey you will be contributing to our knowledge of farm dogs and their handlers. The information you provide will allow us to give the industry an overview of current farm dog usage and will assist with ongoing research.

Completing this survey

Please return this survey in the reply-paid envelope. If you would prefer to complete the on-line version of the survey, it is available at:
http://sydney.edu.au/vetscience/research/animal_behaviour/farmdog/surveys.shtml

You are not obliged to leave your personal details. However, if you would like the opportunity to win a share in over $1,000 worth of Bonnie Working Dog dry food please fill in your contact details when asked. Your identity will be recorded separately from your survey responses.

Section 1. Property

If you have several properties and use working dogs, please answer the questions for the property your dogs most often work on.

What is the size of your property in hectares?

☐Less than 500
☐500 – 1 000
☐1 001 – 3 000
☐3 001 – 7 000
☐7 001 – 15 000
☐15 001 – 30 000
☐More than 30 000

Please indicate the number of each type of livestock on the property.

Cattle

☐Nil
☐Less than 100
☐100 – 500

	☐501 – 1 500	
	☐1 501 – 3 000	
	☐3 001 – 8 000	
	☐More than 8 000	
Sheep	☐Nil	
	☐Less than 500	
	☐500 – 2 000	
	☐2 001 – 5 000	
	☐5 001 – 10 000	
	☐10 001 – 25 000	
	☐More than 25 000	
Other.	☐	

Please specify____

____

In what state is the property?

☐ACT
☐NSW
☐NT
☐QLD
☐SA
☐TAS
☐VIC
☐WA

Section 2. About your dogs

How many dogs do you currently have <u>in work</u>?

☐Nil	☐6
☐1	☐7
☐2	☐8
☐3	☐9
☐4	☐10
☐5	☐More than 10

Please now focus on the dog(s) you work with most often, and answer the following questions. You can submit details for one, two or three dogs. When you have finished answering for the desired number of dogs proceed to **section 3** on page 8**.**

Dog 1

What sex is this dog?

☐Male entire
☐Male desexed
☐Female entire
☐Female desexed

How old is this dog to the closest year?

☐Less than 1	☐7
☐1	☐8
☐2	☐9
☐3	☐10
☐4	☐11
☐5	☐12
☐6	☐Older than 12

What breed is this dog?

☐Kelpie ☐Kelpie cross

☐Border collie ☐Border collie cross
☐Coolie ☐Coolie cross
☐Cattle dog ☐Cattle dog cross
☐Other
Please specify____________________________

What colour is this dog?

☐Black ☐Blue merle
☐Black & tan ☐Brown
☐Black & white ☐Brown & white
☐Red ☐Fawn
☐Red & tan ☐Fawn & tan
☐Red merle ☐Cream (yellow)
☐Blue ☐Chocolate
☐Blue & tan ☐Black, white & tan
☐Other
Please specify_____________________________

What type of work do you mostly use this dog for?

☐Yard (forcing)
☐Mustering
☐Both (all-rounder)
☐Trials <u>only</u>

What stock does this dog work with?
Select all that apply.

☐Sheep
☐Cattle
☐Goats
☐Other. Please specify______________________

How is this dog housed?

☐Individual shelter on chain
☐Individual shelter with yard/pen
☐Individual cage
☐Group cage
☐Group shelter with yard/pen
☐Indoors with humans

What have been the approximate <u>non-routine</u> veterinary costs for this dog in the past five years?

☐$0
☐Less than $500
☐$500 - $2 000
☐More than $2 000

Is this dog insured?

☐Yes ☐No

Where did you acquire this dog?

☐Own breeding program*
☐External breeder
☐Other. Please specify_____________________
____

If this is a **self-bred** dog, please skip the next <u>three</u> questions and continue from the question marked with *.
If acquired from an external source:

What age was it acquired?

☐Less than 8 weeks
☐8 – 12 weeks
☐3 – 6 months
☐Older than 6 months

What level of training did it have when acquired?

☐Unstarted
☐Started
☐Fully trained

How much did you pay for this dog?

☐$0
☐Less than $500
☐$500 - $1 000
☐$1 001 - $2 000
☐$2 001 - $5 000
☐More than $5 000

*Does this dog compete in trials?

☐Yes
☐No

If **no**, proceed to the questions for **Dog 2** or to **section 3** on page 8.

If **yes**:

What type of trials?

☐Utility
☐Yard
☐3-sheep
☐Cattle

In approximately how many trials has the dog competed?

☐1 – 3
☐4 – 6
☐7 – 9
☐10 – 15
☐Over 15

What is the highest division this dog has competed in?

☐Novice
☐Improver
☐Open

What is the highest place they have been awarded in the highest division they have competed?

☐Has not placed
☐First
☐Second
☐Third

If there is a second dog you work with that you would like to give us details on, please continue with **Dog 2.** If not, then proceed to **section 3** on page 8.

Dog 2

What sex is this dog?

☐Male entire
☐Male desexed
☐Female entire
☐Female desexed

How old is this dog to the closest year?

☐Less than 1
☐1
☐2
☐3
☐4
☐5
☐6
☐7
☐8
☐9
☐10
☐11
☐12
☐Older than 12

What breed is this dog?

☐Kelpie
☐Kelpie cross
☐Border collie
☐Border collie cross
☐Coolie
☐Coolie cross
☐Cattle dog
☐Cattle dog cross
☐Other
Please specify_______________________

What colour is this dog?

☐Black
☐Blue merle
☐Black & tan
☐Brown
☐Black & white
☐Brown & white
☐Red
☐Fawn
☐Red & tan
☐Fawn & tan
☐Red merle
☐Cream (yellow)
☐Blue
☐Chocolate
☐Blue & tan
☐Black, white & tan
☐Other
Please specify________________________

What type of work do you mostly use this dog for?

☐Yard (forcing)
☐Mustering
☐Both (all-rounder)
☐Trials <u>only</u>

What stock does this dog work with?
Select all that apply.

☐Sheep
☐Cattle
☐Goats
☐Other. Please specify__________________

How is this dog housed?

☐Individual shelter on chain
☐Individual shelter with yard/pen
☐Individual cage
☐Group cage
☐Group shelter with yard/pen
☐Indoors with humans

What have been the approximate <u>non-routine</u> veterinary costs for this dog in the past five years?

☐$0
☐Less than $500
☐$500 - $2 000
☐More than $2 000

Is this dog insured?

☐Yes
☐No

Where did you acquire this dog?

☐Own breeding program*
☐External breeder
☐Other. Please specify__________________
____

If this is a **self-bred** dog, please skip the next <u>three</u> questions and continue from the question marked with *.
If acquired from an external source:

What age was it acquired?

☐Less than 8 weeks
☐8 – 12 weeks
☐3 – 6 months

	☐Older than 6 months	
What level of training did it have when acquired?	☐Unstarted<br>☐Started<br>☐Fully trained	
How much did you pay for this dog?	☐$0<br>☐Less than $500<br>☐$500 - $1 000	☐$1 001 - $2 000<br>☐$2 001 - $5 000<br>☐More than $5 000
*Does this dog compete in trials?	☐Yes	☐No

If **no**, proceed to the questions for **Dog 3** or to **section 3** on page 8.

If yes:

What type of trials?	☐Utility<br>☐Yard<br>☐3-sheep<br>☐Cattle
In approximately how many trials has the dog competed?	☐1 – 3<br>☐4 – 6<br>☐7 – 9<br>☐10 – 15<br>☐Over 15
What is the highest division this dog has competed in?	☐Novice<br>☐Improver<br>☐Open

What is the highest place they have been awarded in the highest division they have competed?

☐Has not placed
☐First
☐Second
☐Third

If there is a third dog you work with that you would like to give us details on, please continue to **Dog 3.** If not, then proceed to **section 3** on page 8.

Dog 3

What sex is this dog?	☐Male entire<br>☐Male desexed<br>☐Female entire<br>☐Female desexed	
How old is this dog to the closest year?	☐Less than 1<br>☐1<br>☐2<br>☐3<br>☐4<br>☐5<br>☐6	☐7<br>☐8<br>☐9<br>☐10<br>☐11<br>☐12<br>☐Older than 12

What breed is this dog?

☐Kelpie ☐Kelpie cross
☐Border collie ☐Border collie cross
☐Coolie ☐Coolie cross
☐Cattle dog ☐Cattle dog cross
☐Other
Please specify____________________________

What colour is this dog?

☐Black ☐Blue merle
☐Black & tan ☐Brown
☐Black & white ☐Brown & white
☐Red ☐Fawn
☐Red & tan ☐Fawn & tan
☐Red merle ☐Cream (yellow)
☐Blue ☐Chocolate
☐Blue & tan ☐Black, tan & white
☐Other
Please specify_____________________________

What type of work do you mostly use this dog for?

☐Yard (forcing)
☐Mustering
☐Both (all-rounder)
☐Trials only

What stock does this dog work with?
Select all that apply.

☐Sheep
☐Cattle
☐Goats
☐Other. Please specify______________________

How is this dog housed?

☐Individual shelter on chain
☐Individual shelter with yard/pen
☐Individual cage
☐Group cage
☐Group shelter with yard/pen
☐Indoors with humans

What have been the approximate non-routine veterinary costs for this dog in the past five years?

☐$0
☐Less than $500
☐$500 - $2 000
☐More than $2 000

Is this dog insured?

☐Yes ☐No

Where did you acquire this dog?

☐Own breeding program*
☐External breeder
☐Other. Please specify_____________________
____

If this is a **self-bred** dog, please skip the next three questions and continue from the question marked with *.
If acquired from an external source:

What age was it acquired?

☐Less than 8 weeks
☐8 – 12 weeks

☐3 – 6 months
☐Older than 6 months

What level of training did it have when acquired?	☐Unstarted<br>☐Started<br>☐Fully trained	
How much did you pay for this dog?	☐$0<br>☐Less than $500<br>☐$500 - $1 000	☐$1 000 - $2 000<br>☐$2 000 - $5 000<br>☐More than $5 000
*Does this dog compete in trials?	☐Yes	☐No

If **no**, proceed to **section 3.**

If yes:

What type of trials?	☐Utility<br>☐Yard<br>☐3-sheep<br>☐Cattle
In approximately how many trials has the dog competed?	☐1 – 3<br>☐4 – 6<br>☐7 – 9<br>☐10 – 15<br>☐Over 15
What is the highest division this dog has competed in?	☐Novice<br>☐Improver<br>☐Open

What is the highest place they have been awarded in the highest division they have competed?

☐Has not placed
☐First
☐Second
☐Third

Section 3. Breeding

Do you breed working dogs?	☐Yes	☐No

If **no**, proceed to **section 4** on page 9.
If **yes**, complete **section 3.**

Which breed(s)?	☐Kelpie<br>☐Border collie<br>☐Coolie<br>☐Cattle dog<br>☐Other<br>Please specify________________________	☐Kelpie cross<br>☐Border collie cross<br>☐Coolie cross<br>☐Cattle dog cross
What is the purpose of your breeding program?	☐Professional breeding<br>☐Service own needs only	

☐Hobby

What type, or types, of dogs do you aim to produce?

☐Trial (arena)
☐Yard (forcing)
☐Utility (all-rounder)

How many litters do you usually breed each year?

☐Less than 1	☐4
☐1	☐5
☐2	☐More than 5
☐3	

How many stud males do you have in your breeding program?

☐1	☐4
☐2	☐5
☐3	☐More than 5

How many bitches do you have in your breeding program?

☐1	☐4
☐2	☐5
☐3	☐More than 5

What is the closest relative you would be prepared to breed to your bitch?

☐Father
☐Uncle
☐Grandfather
☐Great-grandfather
☐Unrelated dog only

At what age do your pups usually go to new homes?

☐ Less than 8 weeks
☐8 – 12 weeks
☐Older than 12 weeks

What is the average sale price for your pups?

Unstarted:

☐Less than $300
☐$300 - $600
☐More than $600

Started:

☐Not applicable
☐Less than $600
☐$600 - $2 000
☐$2 001 - $4 000
☐More than $4 000

Section 4.Work

At peak times, how much time does your top dog spend working on average?

Each day:

☐Less than 2 hours
☐2 – 4 hours
☐4 – 6 hours
☐More than 6 hours

Each week:

☐1 day
☐2 days
☐3 days
☐4 days
☐5 days
☐6 days
☐7 days

At <u>off –peak</u> times, how often are your dogs exercised (including time spent off-chain or out of cage)?

☐Less than weekly
☐Weekly
☐Twice weekly
☐3 – 5 times each week
☐Daily
☐At least twice daily

When mustering or droving, how do you usually accompany your dog(s)?
Select all that apply.

☐Not applicable
☐On foot
☐Horseback
☐Motorbike
☐Vehicle

Section 5.Outcomes

Have you ever culled or dismissed a working dog before retirement or injury?

☐Yes ☐No

Of the dogs you acquire or retain for work, what percentage become successful working dogs?

☐Less than 50%
☐50 – 64%
☐65 – 79%
☐80 – 99%
☐100%

Dishonourable discharges (dogs dismissed before old age or injury).

Please now focus on the last dog you had <u>in training</u> that you did not retain as a working dog. You will have the opportunity to answer the questions for up to three dogs.
If you have not dismissed any dogs prior to retirement please proceed to **honourable discharge** questions on page 11.

Dog 1

What is the main reason this dog was dismissed?

☐Health problems
☐Inadequate fitness or stamina
☐Lack of working instinct/natural ability
☐Temperament problems
☐Training problems

At what age was this dog dismissed?

☐Less than 3 months
☐3 – 6 months
☐6 – 12 months

☐More than 12 months

What was the destination of the dog after dismissal?

☐Euthanasia
☐Retained for another purpose
☐Rehomed for another purpose

If there is a second dog you have dismissed (dishonourable discharge) that you can answer questions on please continue, otherwise, proceed to **Honourable discharge** questions.

Dog 2

What is the main reason this dog was dismissed?

☐Health problems
☐Inadequate fitness or stamina
☐Lack of working instinct/natural ability
☐Temperament problems
☐Training problems

At what age was this dog dismissed?

☐Less than 3 months
☐3 – 6 months
☐6 – 12 months
☐More than 12 months

What was the destination of the dog after dismissal?

☐Euthanasia
☐Retained for another purpose
☐Rehomed for another purpose

If there is a third dog you have dismissed (dishonourable discharge) that you can answer questions on please continue, otherwise, proceed to **Honourable discharge** questions.

Dog 3

What is the main reason this dog was dismissed?

☐Health problems
☐Inadequate fitness or stamina
☐Lack of working instinct/natural ability
☐Temperament problems
☐Training problems

At what age was this dog dismissed?

☐Less than 3 months
☐3 – 6 months
☐6 – 12 months
☐More than 12 months

What was the destination of the dog after dismissal?

☐Euthanasia
☐Retained for another purpose
☐Rehomed for another purpose

Honourable Discharges (dogs that were retired)

Please answer the following questions for the last <u>successful</u> working dog(s) you had to retire or that ceased work prematurely.
You will have the opportunity to answer the questions for up to three dogs.

Dog 1

Why was the dog retired?

☐Old age
☐Sudden death

☐Injury
☐Illness
☐Loss of interest in work
☐Developed behavioural problem

At what age was this dog retired, to the closest year?

☐1	☐8
☐2	☐9
☐3	☐10
☐4	☐11
☐5	☐12
☐6	☐13
☐7	☐More than 13

What was the dog's destination after retirement?

☐Euthanasia
☐Retained for another use e.g. pet or stud
☐Rehomed
☐Not applicable (deceased)

If there is a second dog you have retired (honourable discharge) that you can answer questions on please continue, otherwise, proceed to **section 6** on page 13.

Dog 2

Why was the dog retired?

☐Old age
☐Sudden death
☐Injury
☐Illness
☐Loss of interest in work
☐Developed behavioural problem

At what age was this dog retired, to the closest year?

☐1	☐8
☐2	☐9
☐3	☐10
☐4	☐11
☐5	☐12
☐6	☐13
☐7	☐More than 13

What was the dog's destination after retirement?

☐Euthanasia
☐Retained for another use e.g. pet or stud
☐Rehomed
☐Not applicable (deceased)

If there is a third dog you have retired (honourable discharge) that you can answer questions on please continue, otherwise, proceed to **section 6** on page 13.

Dog 3

Why was the dog retired?

☐Old age
☐Sudden death
☐Injury
☐Illness
☐Loss of interest in work
☐Developed behavioural problem

At what age was this dog retired, to the closest year?

☐1
☐2
☐3
☐4
☐5
☐6
☐7
☐8
☐9
☐10
☐11
☐12
☐13
☐More than 13

What was the dog's destination after retirement?

☐Euthanasia
☐Retained for another use e.g. pet or stud
☐Rehomed
☐Not applicable (deceased)

Section 6. Training

If you have unstarted dogs, in general, how long does it take you to train them to a competent working standard?

☐I don't obtain unstarted dogs
☐Less than 7 days
☐1 – 4 weeks
☐1 – 3 months
☐3 – 6 months
☐6 – 12 months
☐Over 1 year

If you have started dogs, in general, how long does it take you to train them to a competent working standard?

☐I don't obtain unstarted dogs
☐Less than 7 days
☐1 – 4 weeks
☐1 – 3 months
☐3 – 6 months
☐6 – 12 months
☐Over 1 year

Are your dogs formally assessed (using documentation)?
Select all that apply.

☐No, at no stage
☐Yes, prior to training
☐Yes, during training
☐Yes, at the end of training
☐Yes, during their working career

How much time is spent with the dog during an average training session?

☐I don't have formal training sessions
☐Less than 15 minutes
☐15 – 30 minutes
☐30 – 60 minutes
☐Greater than 1 hour

How many sessions per month do you spend training an individual dog?

☐I don't have formal training sessions
☐Less than 8
☐8 – 15
☐16 – 30
☐More than 30

Which of the following equipment do you use in training?
Select all that apply.

☐Stick/garden rake ☐Flat collar
☐Body harness ☐Check chain
☐Toy/Article/Dummy ☐Clicker
☐Shock (electric) collar ☐Whistle
☐Head harness (Halti) ☐Toy/article
☐Martingale (limited slip collar)
☐Other
Please specify________________________________

What rewards do you use in training?
Select all that apply.

☐No rewards
☐Allowing continued work with stock
☐Food treats
☐Patting
☐Verbal praise

What correction do you use in training?
Select all that apply.

☐No correction
☐Ceasing work with stock
☐Physical correction
☐Verbal correction

How have you obtained your dog training and handling knowledge?
Select all that apply.

☐From experience
☐Books and manuals
☐At dog training schools
☐Completion of a certified course
State the name of the qualification.__________
____

Section 7. Costs

Please estimate the average yearly cost per dog of feeding and routine health care.

☐Less than $400
☐$400 - $800
☐$801 - $1 500
☐More than $1 500

What is the maximum amount you would consider spending on your best working dog to treat it for a serious illness or injury to allow it to return to work?

☐$200 or less
☐$200 - $500
☐$501 - $1 000
☐$1 001 - $2 000
☐$2 001 - $5 000
☐More than $5 000

Section 8. Selection

Do you select working dogs on any physical characteristics?
Select all that apply.

☐No, I don't make choices based on type
☐Body shape/size
Please specify_______________________
☐Head shape/size
Please specify_______________________
☐Ear shape
Please specify_______________________
☐Height
Please specify_______________________
☐Eye colour
Please specify_______________________
☐Coat colour
Please specify_______________________
☐Other
Please specify_______________________

Section 9. Canine Behaviour Traits

Working manoeuvres

While considering an average working dog, please indicate by circling one of the five marks on the scale, how difficult it is to train the following working manoeuvres.
Several of these terms may not be in common usage. Please do not rate in manoeuvres you are not familiar with. They can be left blank.

Cast

Extremely Easy |——|——|——|——| Almost Impossible

Force

Extremely Easy |——|——|——|——| Almost Impossible

Gathering

Extremely Easy |——|——|——|——| Almost Impossible

Cover

Extremely Easy |——|——|——|——| Almost Impossible

Back

Extremely Easy |——|——|——|——| Almost Impossible

Bark

Extremely Easy |——|——|——|——| Almost Impossible

Bite

Extremely Easy |——|——|——|——| Almost Impossible

Heading

Extremely Easy |——|——|——|——| Almost Impossible

Hold

Extremely Easy |——|——|——|——| Almost Impossible

Balance

Extremely Easy |——|——|——|——| Almost Impossible

Drive

Extremely Easy |——|——|——|——| Almost Impossible

Break

Extremely Easy |——|——|——|——| Almost Impossible

Width

Extremely Easy |——|——|——|——| Almost Impossible

Pull

Extremely Easy |——|——|——|——| Almost Impossible

Lift

Extremely Easy |——|——|——|——| Almost Impossible

Draw

Extremely Easy |——|——|——|——| Almost Impossible

Answer the following questions for the type(s) of dog(s) you have the most experience working with. You may answer the same question up to three times if you have experience in multiple areas.

Please indicate by circling one of the five marks on the scale, how valuable you consider the following manoeuvres.

Please mark the box for the first type of dog you are referring to.

1. ☐All-rounder (utility) ☐Mustering ☐Trial(Arena) ☐Yard

Cast

No value|——|——|——|——|Highly valuable

Force

No value|——|——|——|——| Highly valuable

Gathering

No value|—|—|—|—| Highly valuable

Cover

No value|—|—|—|—| Highly valuable

Back

No value|—|—|—|—| Highly valuable

Bark

No value|—|—|—|—| Highly valuable

Bite

No value|—|—|—|—| Highly valuable

Heading

No value|—|—|—|—| Highly valuable

Hold

No value|—|—|—|—| Highly valuable

Balance

No value|—|—|—|—| Highly valuable

Drive

No value|—|—|—|—| Highly valuable

Break

No value|—|—|—|—| Highly valuable

Width

No value|—|—|—|—| Highly valuable

Pull

No value|—|—|—|—| Highly valuable

Lift

No value|—|—|—|—| Highly valuable

Draw

No value|—|—|—|—| Highly valuable

If you have experience with another type of working dog please answer this question again. Otherwise, proceed to the **Working attributes** section on page 20.

Please indicate by circling one of the five marks on the scale, how valuable you consider the following manoeuvres.

Please mark the box for the second type of dog you are referring to.

2. ☐All-rounder (utility) ☐Mustering ☐Trial(Arena) ☐Yard

Cast

No value|————|————|————|————| Highly valuable

Force

No value|————|————|————|————| Highly valuable

Gathering

No value|————|————|————|————| Highly valuable

Cover

No value|————|————|————|————| Highly valuable

Back

No value|————|————|————|————| Highly valuable

Bark

No value|————|————|————|————| Highly valuable

Bite

No value|————|————|————|————| Highly valuable

Heading

No value|————|————|————|————| Highly valuable

Hold

No value|————|————|————|————| Highly valuable

Balance

No value|————|————|————|————| Highly valuable

Drive

No value|————|————|————|————| Highly valuable

Break

No value|————|————|————|————| Highly valuable

Width

No value|————|————|————|————| Highly valuable

Pull

No value|————|————|————|————| Highly valuable

Lift

No value|————|————|————|————| Highly valuable

Draw

No value|————|————|————|————| Highly valuable

If you have experience with another type of working dog please answer this question again. Otherwise, proceed to the **Working attributes** section on page 20.

Please indicate by circling one of the five marks on the scale, how valuable you consider the following manoeuvres.

Please mark the box for the third type of dog you are referring to.

3. ☐All-rounder (utility) ☐Mustering ☐Trial(Arena) ☐Yard

Cast

No value|————|————|————|————| Highly valuable

Force

No value|————|————|————|————| Highly valuable

Gathering

No value|————|————|————|————| Highly valuable

Cover

No value|————|————|————|————| Highly valuable

Back

No value|————|————|————|————| Highly valuable

Bark

No value|————|————|————|————| Highly valuable

Bite

No value|————|————|————|————| Highly valuable

Heading

No value|————|————|————|————| Highly valuable

Hold

No value|————|————|————|————| Highly valuable

Balance

No value|————|————|————|————| Highly valuable

Drive

No value|————|————|————|————| Highly valuable

Break

No value|————|————|————|————| Highly valuable

Width

No value|————|————|————|————| Highly valuable

Pull

No value|————|————|————|————| Highly valuable

Lift

No value|————|————|————|————| Highly valuable

Draw

No value|————|————|————|————| Highly valuable

Working attributes

Please indicate, by circling one of the five marks on the scale, how valuable you consider the following <u>attributes</u>.

Please mark the box for the first type of dog you are referring to.

1. ☐All-rounder (utility) ☐Mustering ☐Trial(Arena) ☐Yard

Shows eye

No value|————|————|————|————| Extremely valuable

Control

No value|————|————|————|————| Extremely valuable

Initiative (includes working independently, keenness, willingness)

No value|————|————|————|————| Extremely valuable

Trainable (includes tractable)

No value|————|————|————|————| Extremely valuable

Intelligent (includes sagacious, brainy, clever)

No value|————|————|————|————| Extremely valuable

Calm

No value|————|————|————|————| Extremely valuable

Firmness (includes strength, power)

No value|——|——|——|——| Extremely valuable

Style of work (includes width)

No value|——|——|——|——| Extremely valuable

Physical suitability (includes stamina, durability)

No value|——|——|——|——| Extremely valuable

Anticipation

No value|——|——|——|——| Extremely valuable

Boldness

No value|——|——|——|——| Extremely valuable

If you have experience with another type of working dog please answer this question again. Otherwise, proceed to the **General attributes** section on page 23.

Please indicate, by circling one of the five marks on the scale, how valuable you consider the following attributes.

Please mark the box for the second type of dog you are referring to.

2. ☐All-rounder (utility) ☐Mustering ☐Trial(Arena) ☐Yard

Shows eye

No value|——|——|——|——| Extremely valuable

Control

No value|——|——|——|——| Extremely valuable

Initiative (includes working independently, keenness, willingness)

No value|——|——|——|——| Extremely valuable

Trainable (includes tractable)

No value|——|——|——|——| Extremely valuable

Intelligent (includes sagacious, brainy, clever)

No value|——|——|——|——| Extremely valuable

Calm

No value|——|——|——|——| Extremely valuable

Firmness (includes strength, power)

No value|——|——|——|——| Extremely valuable

Style of work (includes width)

No value|————|————|————|————| Extremely valuable

Physical suitability (includes stamina, durability)

No value|————|————|————|————| Extremely valuable

Anticipation

No value|————|————|————|————| Extremely valuable

Boldness

No value|————|————|————|————| Extremely valuable

If you have experience with another type of working dog please answer this question again. Otherwise, proceed to the **General attributes** section on page 23.

Please indicate, by circling one of the five marks on the scale, how valuable you consider the following attributes.

Please mark the box for the third type of dog you are referring to.

3. ☐All-rounder (utility) ☐Mustering ☐Trial(Arena) ☐Yard

Shows eye

No value|————|————|————|————| Extremely valuable

Control

No value|————|————|————|————| Extremely valuable

Initiative (includes working independently, keenness, willingness)

No value|————|————|————|————| Extremely valuable

Trainable (includes tractable)

No value|————|————|————|————| Extremely valuable

Intelligent (includes sagacious, brainy, clever)

No value|————|————|————|————| Extremely valuable

Calm

No value|————|————|————|————| Extremely valuable

Firmness (includes strength, power)

No value|————|————|————|————| Extremely valuable

Style of work (includes width)

No value|————|————|————|————| Extremely valuable

Physical suitability (includes stamina, durability)

No value|———|———|———|———| Extremely valuable

Anticipation

No value|———|———|———|———| Extremely valuable

Boldness

No value|———|———|———|———| Extremely valuable

General attributes

Please indicate, by circling one of the five marks on the scale, how much of each trait you would expect to be present in the ideal dog.

You will have the opportunity to answer the question up to three times if you have experience in more than one environment.

Please mark the box for the first type of dog you are referring to.

1. ☐All-rounder (utility) ☐Mustering ☐Trial(Arena) ☐Yard

Excitability

None |———|———|———|———| A very high degree

Trainability

None |———|———|———|———| A very high degree

Motivation & confidence

None |———|———|———|———| A very high degree

Friendliness

None |———|———|———|———| A very high degree

Cautiousness

None |———|———|———|———| A very high degree

If you have experience with another type of working dog please answer this question again. Otherwise, proceed to the **Shyness-boldness** section on page 24.

Please indicate, by circling one of the five marks on the scale, how much of each trait you would expect to be present in the ideal dog.

Please mark the box for the second type of dog you are referring to.

2. ☐All-rounder (utility) ☐Mustering ☐Trial(Arena) ☐Yard

Excitability

None |———|———|———|———| A very high degree

Trainability

None |——|——|——|——| A very high degree

Motivation & confidence

None |——|——|——|——| A very high degree

Friendliness

None |——|——|——|——| A very high degree

Cautiousness

None |——|——|——|——| A very high degree

If you have experience with another type of working dog please answer this question again. Otherwise, proceed to the **Shyness-boldness** section.

Please indicate, by circling one of the five marks on the scale, how much of each trait you would expect to be present in the ideal dog.

Please mark the box for the third type of dog you are referring to.

3. ☐All-rounder (utility) ☐Mustering ☐Trial(Arena) ☐Yard

Excitability

None |——|——|——|——| A very high degree

Trainability

None |——|——|——|——| A very high degree

Motivation & confidence

None |——|——|——|——| A very high degree

Friendliness

None |——|——|——|——| A very high degree

Cautiousness

None |——|——|——|——| A very high degree

Shyness-Boldness

Please indicate, by circling one of the eight marks on the scale, the balance of shyness and boldness that the ideal dog would exhibit.

You can answer the question up to three times if you have experience with more than one type of dog.

Please mark the box for the first type of dog you are referring to.

1. ☐All-rounder (utility) ☐Mustering ☐Trial(Arena) ☐Yard

Very shy |——|——|——|——|——|——|——| Very bold

If you have experience with another type of working dog please answer this question again. Otherwise, proceed to the **section 10**.

Please indicate, by circling one of the eight marks on the scale, the balance of shyness and boldness that the ideal dog would exhibit.

Please mark the box for the second type of dog you are referring to.

2. ☐All-rounder (utility) ☐Mustering ☐Trial(Arena) ☐Yard

Very shy ├───┼───┼───┼───┼───┼───┼───┤ Very bold

If you have experience with another type of working dog please answer this question again. Otherwise, proceed to the **section 10**.

Please indicate, by circling one of the eight marks on the scale, the balance of shyness and boldness that the ideal dog would exhibit.

Please mark the box for the third type of dog you are referring to.

3. ☐All-rounder (utility) ☐Mustering ☐Trial(Arena) ☐Yard

Very shy ├───┼───┼───┼───┼───┼───┼───┤ Very bold

Section 10. About you

What is your age?

☐18 – 29
☐30 – 39
☐40 – 49
☐50 – 59
☐60 – 70
☐Over 70

Are you male or female? ☐Male ☐Female

Please rank the terms below in the order which best describes how you view your working dog(s). In the boxes, place the numbers 1 (most applicable) to 4 (least applicable).

☐As a workplace resource only
☐As employees
☐As workmates
☐As companions

Please select the answer which reflects how strongly you agree or disagree with the following statements.

I see myself as someone who....

has an active imagination.
☐Strongly disagree ☐Disagree ☐Neutral ☐Agree ☐Strongly agree

has few artistic interests.

☐Strongly disagree ☐Disagree ☐Neutral ☐Agree ☐Strongly agree

does a thorough job.
☐Strongly disagree ☐Disagree ☐Neutral ☐Agree ☐Strongly agree

tends to be lazy.
☐Strongly disagree ☐Disagree ☐Neutral ☐Agree ☐Strongly agree

is outgoing, sociable.
☐Strongly disagree ☐Disagree ☐Neutral ☐Agree ☐Strongly agree

is reserved.
☐Strongly disagree ☐Disagree ☐Neutral ☐Agree ☐Strongly agree

is generally trusting.
☐Strongly disagree ☐Disagree ☐Neutral ☐Agree ☐Strongly agree

tends to find fault in others.
☐Strongly disagree ☐Disagree ☐Neutral ☐Agree ☐Strongly agree

gets nervous easily.
☐Strongly disagree ☐Disagree ☐Neutral ☐Agree ☐Strongly agree

is relaxed, handles stress well.
☐Strongly disagree ☐Disagree ☐Neutral ☐Agree ☐Strongly agree

is considerate and kind to almost everyone.
☐Strongly disagree ☐Disagree ☐Neutral ☐Agree ☐Strongly agree

is curious about many different things.
☐Strongly disagree ☐Disagree ☐Neutral ☐Agree ☐Strongly agree

The end

Thank you for completing this survey. If you would like to enter the draw to win a prize, please enter your details below. The terms and conditions of the prize draw are available on-line at: http://sydney.edu.au/vetscience/research/animal_behaviour/farmdog/surveys.shtml

Name:

____

Address:

____

____

____

Email:

____

Telephone Number:

____
